# From Serendipity to Precision: Integrating AI, Multi-Omics, and Human-Specific Models for Personalized Neuropsychiatric Care

**DOI:** 10.3390/biomedicines13010167

**Published:** 2025-01-12

**Authors:** Masaru Tanaka

**Affiliations:** HUN-REN-SZTE Neuroscience Research Group, Hungarian Research Network, University of Szeged (HUN-REN-SZTE), Danube Neuroscience Research Laboratory, Tisza Lajos krt. 113, H-6725 Szeged, Hungary; tanaka.masaru.1@med.u-szeged.hu; Tel.: +36-62-342-847

**Keywords:** precision medicine, artificial intelligence (AI), neuropsychiatric disorders, induced pluripotent stem cells (iPSCs), multi-omics integration, mental health care, machine learning (ML), dynamic systems analysis, biomarkers, personalized medicine

## Abstract

**Background/Objectives**: The dual forces of structured inquiry and serendipitous discovery have long shaped neuropsychiatric research, with groundbreaking treatments such as lithium and ketamine resulting from unexpected discoveries. However, relying on chance is becoming increasingly insufficient to address the rising prevalence of mental health disorders like depression and schizophrenia, which necessitate precise, innovative approaches. Emerging technologies like artificial intelligence, induced pluripotent stem cells, and multi-omics have the potential to transform this field by allowing for predictive, patient-specific interventions. Despite these advancements, traditional methodologies such as animal models and single-variable analyses continue to be used, frequently failing to capture the complexities of human neuropsychiatric conditions. **Summary:** This review critically evaluates the transition from serendipity to precision-based methodologies in neuropsychiatric research. It focuses on key innovations such as dynamic systems modeling and network-based approaches that use genetic, molecular, and environmental data to identify new therapeutic targets. Furthermore, it emphasizes the importance of interdisciplinary collaboration and human-specific models in overcoming the limitations of traditional approaches. **Conclusions:** We highlight precision psychiatry’s transformative potential for revolutionizing mental health care. This paradigm shift, which combines cutting-edge technologies with systematic frameworks, promises increased diagnostic accuracy, reproducibility, and efficiency, paving the way for tailored treatments and better patient outcomes in neuropsychiatric care.

## 1. Introduction

Medical research often relies on two pillars: systematic data collection and the unpredictable nature of serendipity [1,2,3]. While structured data collection provides a solid empirical foundation, many significant medical breakthroughs have occurred by chance [4,5]. For example, Alexander Fleming’s discovery of penicillin resulted from accidental mold contamination, and Wilhelm Röntgen discovered X-rays while experimenting with cathode rays. In psychiatry, serendipitous findings have been particularly impactful, such as the use of lithium for bipolar disorder (BP) and ketamine for depression—both discovered unexpectedly [6,7]. These instances underscore how unplanned observations have historically led to major advancements in medical science [8,9,10,11] (Table 1). In psychiatric treatment, where new therapies are desperately needed, relying on chance is inadequate and risks stagnation [12,13]. To accelerate progress, we must integrate innovative procedures and technologies that streamline research, enhance predictive accuracy, and broaden discovery scopes [14,15,16,17]. However, depending on chance is increasingly inadequate, especially in psychiatric treatment, where new therapies are urgently needed [8,18]. Mental health disorders like depression, anxiety, and BP are rising globally, affecting millions and straining healthcare systems [19,20]. The unpredictability of serendipitous discoveries means that breakthroughs may not happen promptly, potentially leading to stagnation in therapeutic advancements [21,22,23]. Relying solely on chance overlooks the benefits of proactive, systematic exploration using modern scientific tools [21,24,25]. In an era of escalating mental health challenges, there is a pressing need for more efficient and predictable research methodologies to accelerate the development of new treatments [21,22,26].

Integrating innovative procedures and emerging technologies into medical research is essential to address this need. Advanced analytical tools, such as artificial intelligence (AI) and machine learning (ML) algorithms, can analyze vast datasets efficiently, uncovering patterns and correlations that might remain hidden with traditional methods [27,28,29]. For instance, AI can assist in identifying biomarkers for psychiatric disorders by analyzing genetic, neuroimaging, and clinical data, leading to more personalized treatment approaches [30,31,32]. One notable example of this is in oncology, where AI has been used to analyze tumor genetic profiles to guide immunotherapy, significantly improving treatment outcomes for cancers such as melanoma [33,34,35]. Similarly, in cardiology, ML algorithms have enhanced the early detection of atrial fibrillation through wearable devices, allowing timely intervention and reducing the risk of stroke [36,37]. These successes underscore the transformative potential of precision-based methods in other fields and highlight the promise they hold for psychiatry. Adaptive trial designs allow modifications based on interim results without compromising integrity, making clinical studies more flexible and cost-effective [38,39,40]. Interdisciplinary collaboration brings together experts from neuroscience, genetics, pharmacology, computer science, and bioinformatics, fostering a holistic approach to problem-solving and yielding more robust solutions [41,42,43].

Emerging strategies like induced pluripotent stem cells (iPSCs) and organoids offer human-specific models for deeper insights into disease mechanisms at the cellular level [44,45,46]. iPSCs derived from patients can be differentiated into various cell types, enabling researchers to study disease pathology and test potential treatments in environments closely mimicking human biology [47,48,49]. Organoids—three-dimensional cell cultures replicating organ structures—allow the examination of complex interactions within human tissues [46,50,51]. These models overcome the limitations of traditional animal studies, which often lack relevance to human biology and fail to capture the complexity of human disease interactions.

Multi-omics approaches—integrating genomics, transcriptomics, proteomics, metabolomics, and other ‘omics’ data—provide a comprehensive understanding of biological systems and disease processes [52,53,54]. By analyzing multiple layers of biological information simultaneously, researchers can identify novel therapeutic targets and biomarkers with greater precision [55,56,57]. AI tools further enhance precision and reproducibility by automating data analysis, reducing human error, and handling complex, high-dimensional datasets [53,58,59]. Network-based modeling reveals intricate interactions within biological systems, identifying pathways critical for precision medicine. These models simulate how alterations in one system component affect others, offering insights into disease mechanisms and potential interventions [52,54,58]. An illustrative case is in diabetes management, where the precision modeling of glucose–insulin dynamics has informed personalized insulin therapy, improving blood sugar control and reducing complications [60,61,62]. Similarly, in rheumatoid arthritis, biomarkers have been used to predict patient response to biologic treatments, ensuring that individuals receive the most effective therapy with minimal delay [63,64,65]. These applications highlight how integrating system-level insights can optimize both diagnosis and treatment strategies.

Despite these advancements, a significant research gap remains due to continued reliance on traditional models and serendipity [21,66,67]. One major obstacle in adopting precision-based approaches in neuropsychiatric research is the complexity and heterogeneity of psychiatric disorders themselves [68,69,70]. Unlike diseases with clear biological markers, psychiatric conditions often manifest through a combination of genetic, environmental, and behavioral factors, making it challenging to identify universally applicable biomarkers [71,72,73]. Additionally, the lack of standardized protocols for integrating multi-omics data with clinical phenotypes creates inconsistencies in research outcomes [73,74,75]. Another barrier lies in the ethical and logistical challenges associated with large-scale data collection [75,76,77]. Privacy concerns, particularly when dealing with sensitive neuroimaging and genetic data, hinder widespread adoption [76,78,79]. Moreover, ensuring diverse representation in datasets is critical but often difficult, as many studies disproportionately include participants from developed regions [78,80,81]. Addressing these challenges will require robust frameworks for data sharing, advances in explainable AI to interpret complex datasets, and international collaboration to ensure findings are globally relevant and equitably implemented [75,76,79]. Conventional research paradigms often isolate single variables, failing to account for the dynamic interactions between genes, proteins, and the environment that characterize complex diseases like psychiatric disorders [67,82,83]. Dynamic systems analysis contributes by tracking temporal changes in disease progression, providing predictive insights to tailor interventions more effectively [67,82,84]. Understanding how diseases evolve over time enables clinicians to develop treatment plans that address specific patient needs at different stages [66,85].

Given these challenges, it is imperative for the research community to embrace innovation as a necessity rather than an option. This review aims to highlight the importance of emerging strategies in transforming medical research—particularly in psychiatry—into a field driven by design rather than chance. We will explore how adopting innovative technologies and collaborative approaches can foster an ecosystem where breakthrough treatments are discovered more predictably and efficiently. By emphasizing predictive accuracy, efficiency, and human relevance, we can accelerate the discovery of new treatments and ultimately improve patient outcomes. The review will focus on evaluating the limitations of traditional models and the continued reliance on serendipity, exploring the potential of emerging technologies like iPSCs, organoids, multi-omics, and AI in revolutionizing psychiatric research. It will identify research gaps that hinder the efficient discovery of new therapies and a comprehensive understanding of complex disease mechanisms. Additionally, the review will propose integrative strategies to incorporate innovative procedures and interdisciplinary collaboration into current research frameworks while addressing the ethical considerations and policy changes necessary to support these advancements responsibly. By systematically examining these areas, we aim to provide a roadmap for transitioning from chance-driven discoveries to deliberate, design-focused research. This shift is essential for meeting the urgent need for new psychiatric treatments and enhancing the overall effectiveness of medical research. Embracing these innovations will not only reduce our dependence on serendipity but also pave the way for more predictable and efficient discoveries of breakthrough therapies, ultimately improving outcomes for patients worldwide.

**Table 1:** Historical serendipity in drug discovery for mental illnesses. BP, bipolar disorder; SCZ, schizophrenia; N/A: Not applicable.

Year	Drug Name	Primary Targets	Expected Diseases to Treat	Mental Illnesses Treated	Ref.
1940s–1950s	Iproniazid	Monoamine Oxidase	Tuberculosis	Depression	[9,22]
1950s	Lithium	Unknown	N/A	BP	[8]
1950s	Chlorpromazine	Dopamine Receptors	Sedation	SCZ	[10,22,86]
1950s	Imipramine	Serotonin/Norepinephrine Reuptake	N/A	Depression	[9,22]
1950s	Chlordiazepoxide	GABA Receptors	N/A	Anxiety	[22]
1960s	Psilocybin	Serotonin Receptors	N/A	Depression	[10]
2000s	Ketamine	NMDA Receptors	Anesthesia	Depression	[9,10,11]
2010s	Minocycline	Unknown	Infection	SCZ	[10]
2010s	Warfarin	Blood Clotting Factors	Blood Clotting Disorders	SCZ	[10]

## 2. Integrative Models of Wet and Dry Research

**Figure 1:**
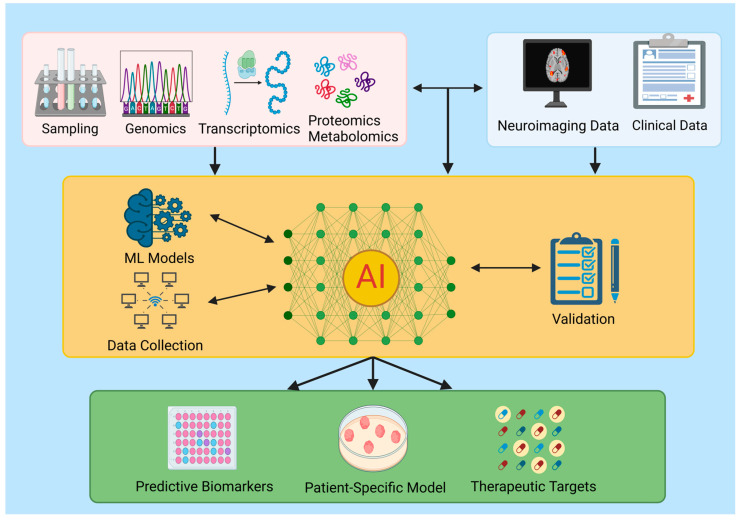
Multi-omics and artificial intelligence (AI) integration for neuropsychiatric precision medicine. A comprehensive framework for training AI-powered machine learning models by integrating multi-omics data (genomics, transcriptomics, proteomics, and metabolomics), neuroimaging data, and clinical information. The process entails the use of machine learning (ML) models, data collection, and validation trough model training, which allows for the identification of predictive biomarkers, the creation of patient-specific models, and the discovery of novel therapeutic targets.

The integration of wet and dry research is crucial for advancing treatments of neuropsychiatric disorders. Wet research, involving experimental and clinical studies, provides empirical data, while dry research, encompassing computational models and data analysis, offers predictive insights [87,88,89,90,91,92]. Combining these approaches enhances the understanding of complex neuropsychiatric conditions and improves treatment strategies (Figure 1) [88,93,94].

In cardiac research, integrating experimental data into computational models has allowed researchers to refine treatments and predicted outcomes [95]. Similarly, partnerships between AI and neurology have advanced neuroimaging biomarkers for Alzheimer’s disease (AD), enabling earlier and more accurate diagnoses [95,96,97,98]. The triadic relationship between vascular dysfunction, muscle atrophy, and cognitive decline underscores the necessity for multidisciplinary approaches that address these interconnected mechanisms [99,100,101]. For instance, integrative medicine approaches have shown promise in treating post-stroke depression by combining traditional Chinese medicine, Western medicine, and rehabilitation techniques, leading to improved patient outcomes [95,102]. Integrative psychotherapy models for conditions like psychogenic nonepileptic seizures and anxiety disorders have demonstrated significant efficacy by incorporating cognitive behavioral techniques, psychoeducation, and individualized treatment protocols [103,104,105]. Furthermore, integrative care models for Parkinson’s disease and AD emphasize multidisciplinary approaches, combining pharmacotherapy with allied health therapies to effectively manage both motor and neuropsychiatric symptoms [106,107,108,109,110]. These examples underscore the necessity of integrative approaches that leverage both empirical data from wet research and predictive models from dry research to develop comprehensive treatment plans, ultimately enhancing patient care in neuropsychiatric disorders. iPSC technologies are valuable for modeling disease mechanisms and testing potential treatments in vitro, but they are limited by high costs, labor intensity, and expertise requirements, highlighting the need for automation and cost reduction [108,111,112,113]. AI predictions, although promising, face validation issues due to biases, limited generalizability, and opacity, necessitating diverse datasets, explainable AI, and multi-site validation [111,114,115].

Wet research employs advanced techniques like genome-wide association studies (GWAS) to identify the genetic loci associated with neuropsychiatric disorders, providing insights into their genetic basis [116,117,118]. The integration of wet and dry research has proven effective in fields such as cardiac mechano-electric function studies, where experimental data are used to build and validate computational models, enhancing our understanding of cardiac behavior [119,120,121]. In toxicology, combining high-throughput wet lab techniques with computational methods addresses the challenges of analyzing high-dimensional data, translating complex data into actionable insights. Innovative educational programs are also incorporating both wet and dry lab experiences, such as using CRISPR/Cas9 for gene editing in mouse stem cells alongside computer simulations to generate transgenic mouse models, enriching learning and reducing animal testing.

Computational models play a pivotal role in dry research by integrating and analyzing extensive datasets from wet research. They are essential for understanding complex biological systems and predicting the effects of various factors [122,123]. Systems-level integrative pathway analyses have been instrumental in elucidating the polygenic contributions of risk variants to neuropsychiatric disorders, guiding the development of targeted therapies [123,124,125,126]. Computational models in cardiac research have evolved over decades, enhancing our understanding of cardiac function and predicting outcomes [127,128,129]. Similarly, computational fluid dynamics has revolutionized the modeling of drying processes, optimizing technologies across multiple scientific domains [130,131].

## 3. Cyclic Data Processing

To provide a multidimensional view of biological systems and disease mechanisms, the cyclic data processing framework begins with the systematic collection of various data types, including genetic, epigenetic, transcriptomic, proteomic, and clinical datasets [132,133,134]. Integrative multi-omics approaches, such as combining GWAS with epigenetic and transcriptomic data, facilitate the identification of novel genetic loci and potential therapeutic targets [135,136]. For example, integrating single-cell RNA sequencing with chromatin accessibility data has revealed cell-type-specific regulatory elements in neuropsychiatric disorders, which is critical for understanding complex diseases like schizophrenia (SCZ) or BP [116,136,137,138,139].

ML and statistical methods are used to create predictive models from integrated datasets. These models enable the forecasting of disease progression, patient stratification, and treatment outcomes [140,141]. To ensure clinical reliability, these predictions undergo rigorous validation through experimental techniques such as CRISPR-based functional genomic studies or the in vitro study of neural organoids derived from patient-specific iPSCs [140,142]. This iterative process of prediction and validation refines models and enhances their clinical applicability, advancing precision medicine [140,142,143].

The transition from micro to macro in cyclic data processing allows for breakthroughs in complex biological systems, connecting molecular insights to large-scale applications [144,145]. Micro-level research focuses on fundamental molecular and cellular mechanisms, such as the role of G protein-coupled receptors (GPCRs) and their modulation in neuropsychiatric disorders [144,146,147,148]. These receptors are crucial in neurotransmission, offering potential for targeted therapeutic interventions. Similarly, epigenetic mechanisms like histone modification and non-coding RNA (ncRNA) regulation provide insight into how cellular processes adapt to environmental changes [149,150]. The dysregulation of ncRNAs, such as microRNAs, which regulate gene expression and neural plasticity, has been linked to conditions such as SCZ and depression. Therapies aimed at ncRNAs, such as microRNA mimics, show promise in modulating synaptic function and neuroinflammation.

Understanding emergent properties and using advanced computational tools such as ML to model system-wide effects are required for translating these findings into macro-level applications [151,152,153,154,155]. Integrating genomic and proteomic data with deep clinical phenotyping has enabled the development of precision medicine strategies [156,157,158,159]. Patient-specific models derived from iPSCs are used to simulate disease progression and test therapeutic responses [160,161,162]. This strategy has been used in oncology, where genetic profiling informs targeted treatments, and in neurodegenerative diseases such as AD, where cellular models predict patient-specific drug efficacy [160,162,163,164,165,166,167].

To summarize, the cyclic data processing framework connects micro-level molecular insights to macro-level applications by integrating diverse datasets and predictive modeling. This approach promotes a thorough understanding of complex diseases and advances precision medicine, allowing for the development of targeted therapies for neuropsychiatric and other complex disorders.

## 4. Interpreting Experimental Results

Interpreting experimental results in neuropsychiatric research is challenging due to the complexity of these disorders. Animal models, while valuable, cannot fully replicate human conditions, necessitating cautious interpretation and validation in human models [168,169,170]. Overreliance on statistical significance, particularly P values, can lead to misinterpretations; treating nonsignificant results as evidence of no effect confuses the absence of evidence with evidence of absence [171,172]. Variability in diagnostic accuracy using different interpretive approaches can yield inconsistent outcomes [173,174]. The complexity of neuroimaging data adds further challenges [174,175]. ML-based predictive models in neuroimaging frequently lack interpretability and require extensive validation across multiple datasets to ensure reliability [176,177,178]. Presenting only significant results can obscure the full picture, leading to biases and reproducibility issues [173,179,180]. Furthermore, AI-powered neuroimaging analyses may introduce bias if algorithms are trained on non-representative datasets, reducing clinical utility [178,179]. The use of sensitive imaging and genomic data necessitates stringent privacy protections (Table 2) [181,182,183]. As a result, a comprehensive approach—including careful statistical analysis, validation in human models, and transparent reporting—is required for accurate interpretation in neuropsychiatric research.

Translational research bridges the gap between experimental findings and clinical applications by converting laboratory discoveries into practical treatments [184,185]. This process is crucial for developing effective therapies for diseases like neuropsychiatric disorders. For example, novel therapies targeting neuroinflammatory pathways in glial cells are being investigated using insights from iPSC-derived models [171,186,187]. Integrating high-throughput experimental data with existing knowledge and automated inference tools, as seen in GWAS, demonstrates the power of translational research frameworks [188,189,190]. Ensuring robustness across genetically diverse populations improves the translational potential of preclinical findings, leading to a better prediction of treatment responses in heterogeneous patient groups [191,192]. The blinded interpretation of study results reduces bias and enhances reliability [191,193]. Translational research, aided by initiatives such as the National Institutes of Health’s Center for Advancing Translational Sciences, emphasizes the importance of collaborative efforts among researchers, clinicians, and funders in effectively translating laboratory findings into clinical applications [194,195,196]. By addressing uncertainties and ensuring rigorous, reproducible methodologies, translational research continues to play a pivotal role in advancing medical science and improving patient care [197,198].

## 5. Towards Patient-Specific Models

Precision medicine is the future of neuropsychiatric disorder treatment, as it combines genetic, clinical, and environmental data to create patient-specific models that predict disease risk and treatment response [199,200,201]. This personalized approach aims to improve patient outcomes by tailoring care to individual needs [200,202,203]. To find underlying biological drivers and enable targeted drug development in neuropsychiatric disorders, precision medicine uses patient stem cell models, deep clinical phenotyping, and genomics [56,204]. These conditions require thorough functional genomic annotation and experimental validation using in vivo or in vitro model systems due to their highly polygenic and pleiotropic nature [57].

Environmental and socioeconomic factors like stress, diet, and access to care significantly affect neuropsychiatric outcomes [205,206]. Including these factors in predictive models enhances accuracy and addresses health disparities, enabling more personalized interventions. For example, in SCZ, precision medicine involves using biological markers to individualize treatment, predict future illness, and determine outcomes over the disease course [202,207,208,209]. Precision clinical trials for neurobehavioral disorders use adaptive treatments and precise measurement techniques to improve personalized care [210]. In epilepsy, precision medicine extends beyond genetics to include a broader array of personalized factors, aiming to address both seizures and associated comorbidities [202,211].

AI and ML have the potential to transform neuropsychiatry by predicting disease progression, aiding patient stratification, and identifying biomarkers [210,212]. However, challenges such as overfitting due to limited datasets, biases in training data, and a lack of interpretability hinder clinical adoption [213,214,215]. These issues highlight the need for explainable AI frameworks, diverse datasets, and rigorous validation to ensure reliable and equitable applications.

Clinical trials and case studies are required to validate patient-specific models. Integrative psychotherapy models for psychogenic nonepileptic seizures have demonstrated promising outcomes in terms of seizure frequency reduction and improved patient functioning [216]. Patient-derived xenograft models have been used in clinical trials to evaluate the efficacy of anticancer drugs, providing a strong foundation for personalized cancer treatment [216]. Patient-specific computational models in congenital heart disease have aided in planning medical procedures and predicting clinical outcomes [216]. Involving patients and the public in clinical trials improves study design, recruitment, and communication, enhancing the relevance and impact of research [216,217].

Developing patient-specific models requires balancing the use of detailed personal data with ethical considerations [218,219,220]. Privacy concerns must be addressed through transparent consent processes and secure data management systems [218,221,222]. Furthermore, biases in computational frameworks may impede the equitable implementation of precision medicine, emphasizing the importance of algorithms that are both accurate and fair across diverse patient populations [223,224].

## 6. Discussion

The field of neuropsychiatric research stands at a critical crossroads, navigating between traditional methodologies and the burgeoning potential of precision-based approaches [71,225]. Historically, many significant advances in this domain have emerged serendipitously, driven by unexpected discoveries [21,226]. Examples such as the therapeutic use of lithium for BP and the antidepressant effects of ketamine underscore the transformative impact of chance findings [225,226,227,228]. These breakthroughs, while revolutionary, often came at the expense of time and systematic predictability [21,229]. Serendipity, by its very nature, lacks reproducibility and scalability, limiting its ability to address the rapidly growing global burden of mental health disorders [10,21,230]. Disorders like depression, anxiety, and SCZ are increasing in prevalence, necessitating more reliable and efficient strategies to uncover effective treatments [13,16,231]. In this context, the limitations of serendipitous discoveries have become apparent, prompting the research community to seek innovative methods that align with the demands of modern medicine [22,232,233].

The shift from serendipity to precision-based approaches represents a paradigm change in neuropsychiatric research [71,234,235]. Precision medicine emphasizes tailored treatments, leveraging patient-specific data to improve diagnostic accuracy and therapeutic outcomes [201,236,237]. This approach builds on advancements in technologies such as AI, iPSCs, and multi-omics integration [234,237,238]. For instance, in cystic fibrosis, precision medicine has revolutionized treatment through the identification of genetic mutations like *F508del*, enabling the development of targeted therapies such as cystic fibrosis transmembrane conductance regulator (CFTR) modulators [239,240,241]. These treatments have dramatically improved lung function and quality of life for patients with specific genetic profiles. In hematology, genomic analysis has allowed for the precise classification of leukemia subtypes, guiding personalized chemotherapy regimens that enhance survival rates [242,243]. These examples illustrate how precision-based approaches have redefined therapeutic paradigms in diverse areas of medicine and emphasize their potential applicability to neuropsychiatric disorders. The innovations enable researchers to identify disease mechanisms at unprecedented levels of detail, offering insights into complex biological interactions [71,235,244]. The evolution toward precision is not merely a technological shift: it reflects a broader commitment to systematic, reproducible, and predictive science [234,245,246]. By transitioning to data-driven methodologies, the field aims to replace chance with design, fostering an era of intentional discovery and targeted intervention [247,248,249]. This evolution underscores the urgency of integrating cutting-edge tools to address the challenges of neuropsychiatric disorders effectively [250,251,252].

A paradigm shift in psychiatric research calls for moving beyond traditional, primarily categorical diagnostic systems toward frameworks grounded in neurobiology and observable behaviors [253,254,255]. While Diagnostic and Statistical Manual of Mental Disorders, Fifth Edition (DSM-5) and International Classification of Diseases, Eleventh Revision (ICD-11) predominantly rely on categorical classifications (with DSM-5 incorporating only minimal dimensional features), the Research Domain Criteria (RDoC) framework and the Hierarchical Taxonomy of Psychopathology (HiTOP) adopt a dimensional, integrative approach to understanding mental health [256,257,258]. Rather than grouping disorders solely by clinical symptom clusters, RDoC and HiTOP focus on core domains of functioning—such as cognition, emotion, and arousal—linked to measurable biological constructs, behavioral data, and neural circuits [258,259]. For instance, the RDoC’s cognitive systems domain examines processes like attention and memory, illuminating the mechanisms that bridge symptoms and underlying neurobiology [260,261,262]. Incorporating these dimensional frameworks into research and diagnostic practices has the potential to unify disparate approaches, reduce heterogeneity in patient populations, and improve reproducibility across studies [253,261,263]. Ultimately, RDoC and HiTOP offer a scientifically grounded path to personalized interventions, tailoring treatments to individual biological and behavioral profiles [263,264,265].

This review highlights the transformation of neuropsychiatric research, emphasizing the transition from traditional, chance-driven discoveries to deliberate, precision-based methodologies. This paper outlines the limitations of conventional approaches, such as serendipitous findings and animal models, which often fail to capture the complexity of human neuropsychiatric conditions. In response, it underscores the necessity of integrating advanced technologies and interdisciplinary methods to uncover novel therapeutic targets and improve patient outcomes. Key insights include the importance of dynamic systems analysis, which tracks temporal changes in disease progression, and network-based modeling, which identifies critical biological pathways. By focusing on predictive and personalized strategies, this review positions precision medicine as the cornerstone of future neuropsychiatric research, aiming to achieve greater accuracy, reproducibility, and efficiency in treatment development.

This review also details the integration of transformative technologies that are reshaping the field. AI and ML provide unparalleled capabilities for analyzing large, complex datasets, uncovering patterns that traditional methods often overlook. iPSCs and organoids offer human-specific models to study disease mechanisms and test potential therapies in environments that closely mimic human biology. Multi-omics approaches combine genomics, transcriptomics, proteomics, and metabolomics to deliver a comprehensive view of disease processes, enabling the identification of biomarkers and therapeutic targets with precision. Collectively, these innovations represent a unified framework for advancing neuropsychiatric research, bridging gaps between basic science, translational studies, and clinical applications. This review underscores the synergistic potential of these tools in addressing the unmet needs of neuropsychiatric disorders.

The ultimate goal of neuropsychiatric research is to transition from generalized, trial-and-error treatment approaches to predictive, patient-specific treatments tailored to individual biological, environmental, and clinical profiles [71,235,236]. This shift aligns with the broader objectives of precision medicine, which seeks to enhance therapeutic efficacy and minimize adverse effects by accounting for the unique characteristics of each patient [244,266,267]. In neuropsychiatric care, where disorders like depression, BP, and SCZ are heterogeneous and multifaceted, this approach holds transformative potential [116,268,269]. Patient-specific treatments can better address the diverse manifestations of these disorders, which are often influenced by genetic predispositions, environmental exposures, and lifestyle factors [270,271,272]. Predictive tools such as biomarkers, advanced imaging, and personalized diagnostic algorithms offer the promise of identifying at-risk individuals and intervening early, potentially altering the trajectory of illness and improving quality of life (Table 3) [273,274,275].

The early detection of psychiatric disorders, including SCZ, BP, and depression, is fundamental to improving outcomes and advancing precision medicine [32,297,298]. Biomarkers derived from neuroimaging, genetics, and multi-omics data are particularly valuable, as they reveal biological and physiological changes that often occur long before clinical symptoms appear [32,298,299]. For instance, subtle shifts in brain volume or abnormal connectivity patterns observed through functional magnetic resonance imaging (fMRI) have been associated with heightened risk for these conditions [40,300,301]. Similarly, genetic variations can indicate a predisposition to mental health issues, making these tools indispensable in identifying at-risk individuals [302,303,304]. Behavioral changes are another critical component of early diagnosis [302,304]. Sleep disturbances, cognitive deficits, and emotional dysregulation frequently emerge during the initial stages of psychiatric disorders [302,303,304]. In the case of SCZ, mild hallucinations, social withdrawal, or declining functionality often signal the prodromal phase, yet these symptoms frequently go unnoticed [304,305,306]. Combining behavioral insights with biological markers enhances diagnostic precision, enabling timely interventions to alter disease trajectories and improve quality of life [302,304,306].

AI is transforming the landscape of early psychiatric diagnosis by introducing unprecedented analytical capabilities [307,308]. Unlike traditional methods, AI-powered models can process large, multi-modal datasets to uncover intricate patterns among biomarkers, behaviors, and environmental factors [308,309]. These tools are especially effective in identifying subtle changes that might otherwise be overlooked [310,311,312]. For example, ML algorithms have demonstrated remarkable success in predicting the onset of psychosis by analyzing speech patterns, neuroimaging findings, and genetic data [309,310,313]. The implications of early intervention extend beyond individual health benefits. Preventive strategies, such as cognitive behavioral therapy or pharmacological treatments administered during the prodromal phase, can delay or mitigate the progression of psychiatric conditions [308,314]. This, in turn, reduces hospitalizations, alleviates the strain on healthcare systems, and lowers associated societal costs [292,315]. AI-driven insights, when combined with interdisciplinary collaboration and a focus on prevention, represent a paradigm shift [292].

Integrating objective measures such as biomarkers and structured frameworks like the RDoC into psychiatric diagnostics could transform the field by standardizing diagnostic criteria [253,254,263,316,317,318]. Current methods, which often rely on subjective clinical observations and self-reports, introduce variability, which hampers research cohesion and clinical reproducibility [32,254,260,319,320]. By contrast, biomarkers provide measurable and consistent data points that capture the underlying biological mechanisms of disorders, reducing ambiguity in diagnosis [32,260,318,319,320]. Standardized diagnostic criteria would create more homogeneous research cohorts, ensuring that studies are conducted on well-defined and comparable patient populations [253,254,260,263,316,317]. This uniformity would enhance the reproducibility of findings across different studies and improve the translatability of preclinical and clinical research into practical treatments [321,322,323]. Moreover, it would enable a more accurate stratification of patients for targeted therapies, paving the way for precision medicine in psychiatry [201,202,324]. A shift toward such integrative, biomarker-driven approaches holds the potential to unify the field, addressing the long-standing challenges of heterogeneity and variability in psychiatric research and improving outcomes for patients globally [201,325,326].

Precision methodologies are vital to realizing this goal, as they enable a deeper understanding of complex neuropsychiatric conditions [71,201,235]. Traditional diagnostic methods and treatment paradigms often fail to capture the nuanced interplay of genetic, molecular, and environmental factors, resulting in variable outcomes and limited progress [327,328,329]. Precision approaches leverage cutting-edge technologies, including multi-omics, AI, and patient-derived models like iPSCs [330,331,332]. By integrating these tools, researchers can identify specific disease mechanisms, predict individual responses to therapies, and tailor interventions with greater accuracy. The necessity of these methodologies is underscored by the rising prevalence and societal impact of neuropsychiatric disorders, which demand innovative strategies to address unmet clinical needs [333,334,335,336].

The transition to predictive, patient-specific treatments is hindered by several challenges, including the limitations of traditional models and reliance on serendipity. Historically, many neuropsychiatric therapies have emerged unexpectedly, highlighting the unpredictability of chance-driven discoveries [16,337,338]. While such breakthroughs have been valuable, they often lack the scalability and reproducibility required to address modern healthcare demands [16,339,340]. Conventional research methods, particularly those relying on animal models, fail to adequately mimic human neuropsychiatric conditions, limiting their translational value [338,341,342]. These limitations underscore the need for human-specific models and systematic, hypothesis-driven approaches that prioritize reproducibility and precision. In addition to methodological challenges, there are significant gaps in knowledge and infrastructure. The complex interplay of genetic, molecular, and environmental factors in neuropsychiatric disorders remains poorly understood, impeding the development of targeted interventions [271,272,343]. The insufficient integration of interdisciplinary expertise further hinders progress, as effective solutions require collaboration among neuroscientists, geneticists, data scientists, and clinicians [344,345,346]. Infrastructure challenges include limited access to advanced technologies, fragmented datasets, and the lack of standardized frameworks for data sharing and analysis [346,347,348]. Addressing these gaps is crucial for building a robust foundation for precision neuropsychiatry.

Achieving the goal of predictive, patient-specific neuropsychiatric care necessitates the integration of essential innovations such as AI, ML, and multi-omics. AI and ML technologies are transformative in their ability to process and analyze large, complex datasets, uncovering patterns and relationships that traditional methods cannot [244,349,350]. These tools are instrumental in identifying biomarkers, stratifying patients, and predicting treatment outcomes with unprecedented accuracy [214,349,351]. Multi-omics approaches—combining genomics, transcriptomics, proteomics, and metabolomics—provide a comprehensive understanding of the molecular underpinnings of neuropsychiatric disorders [298,352,353]. Together, these technologies enable the development of precise, individualized interventions. Dynamic systems analysis and network-based modeling are critical for understanding the intricate interactions within biological systems. Dynamic systems analysis captures temporal changes in disease progression, offering insights into the timing and efficacy of interventions [354,355,356]. Network-based modeling reveals the complex relationships between genes, proteins, and environmental factors, identifying key pathways and nodes that can serve as therapeutic targets [354,357,358]. These approaches shift the focus from isolated components to holistic, system-level insights, providing a more accurate representation of disease mechanisms. The successful application of these technologies requires a supportive research ecosystem. This includes access to diverse, high-quality datasets, collaboration across disciplines, and investments in training programs to equip researchers with the skills needed to utilize these tools effectively. By addressing these technological and knowledge requirements, the field of neuropsychiatry can move closer to achieving its ultimate goal of predictive, patient-specific care.

Advancing neuropsychiatric research is crucial for addressing the global mental health crisis, as disorders such as depression, anxiety, and SCZ rank among the leading causes of disability worldwide [334,359]. These conditions impose substantial social and economic burdens, yet traditional diagnostic and treatment methods often fall short of addressing their complexity [360,361,362]. Precision psychiatry provides a transformative solution by tailoring care to individual biological, genetic, and environmental profiles [363,364,365]. This approach enhances diagnostic accuracy, enables early interventions, and optimizes therapeutic outcomes, shifting the focus from generalized treatments to personalized care [187,362,363]. Technologies like multi-omics and AI drive this transition, identifying biomarkers and predicting treatment responses with unprecedented precision. Beyond innovation, this shift fulfills an ethical imperative to provide equitable and effective healthcare. By addressing these challenges through precision psychiatry, the field can significantly reduce the burden of mental health disorders and improve patient outcomes.

This review synthesizes recent advancements in neuropsychiatric research, integrating technologies like AI, multi-omics, and patient-derived models such as iPSCs and organoids. It builds on prior frameworks, which often relied on serendipity or animal models that lack human-specific relevance and scalability. By bridging traditional methodologies with contemporary approaches, this review outlines a roadmap for precision-based research and therapeutic strategies. It highlights the importance of interdisciplinary collaboration and robust infrastructure to support these innovations. By situating these advancements in the broader scientific context, this review demonstrates how emerging tools can overcome historical limitations, paving the way for transformative breakthroughs in neuropsychiatric care.

Precision psychiatry has profound clinical implications, driven by AI-powered diagnostics and personalized interventions. AI and ML can identify complex patterns in patient data, enhancing diagnostic accuracy and predicting treatment outcomes. These tools enable personalized care plans tailored to individual needs, improving therapeutic efficacy while minimizing side effects. Meanwhile, patient-derived iPSCs and organoids provide human-specific models to study disease mechanisms and test therapies, mimicking biological conditions with exceptional fidelity. Together, these innovations herald a new era of targeted, efficient, and effective mental healthcare, addressing unmet clinical needs and transforming patient outcomes.

This review’s key strength lies in its comprehensive integration of technological and biological insights, forming a robust foundation for advancing neuropsychiatric research. By synthesizing innovations such as AI, multi-omics, and patient-derived models like iPSCs and organoids, it highlights tools addressing long-standing challenges in understanding and treating complex disorders. Additionally, the inclusion of dynamic systems analysis and network-based modeling demonstrates the potential for uncovering intricate disease mechanisms, offering a system-level perspective on neuropsychiatric conditions. This emphasis on human-specific models bridges critical gaps left by traditional animal models and serendipitous findings. This review also underscores the importance of multidisciplinary approaches, emphasizing collaboration across neuroscience, genetics, bioinformatics, and clinical psychiatry. This cross-disciplinary focus is crucial for tackling the complexity of mental health disorders, which demand diverse expertise. Furthermore, this review provides actionable strategies for integrating advanced technologies into clinical and research frameworks, offering a roadmap for implementing precision psychiatry. By combining theoretical insights with practical directions, it serves as a valuable resource for advancing the field. By synthesizing advanced methodologies and promoting interdisciplinary collaboration, this review not only addresses existing gaps in neuropsychiatric research, but also sets the stage for transformative breakthroughs, benefiting researchers, clinicians, and patients alike.

## 7. Outlook

Future research in neuropsychiatric disorders should prioritize refining integrative models and fostering collaboration between experimental (“wet”) and computational (“dry”) labs. By combining computational modeling, AI, multi-omics, and experimental methods like CRISPR technology, researchers can advance precision medicine. Interdisciplinary training programs that merge ML with experimental techniques prepare scientists to tackle complex neuropsychiatric challenges. Techniques such as single-cell sequencing and deep learning in neuroimaging can identify cell-type-specific mechanisms and biomarkers in disorders like SCZ and autism, leading to more precise therapeutic targets [366]. Interpretability tools enhance clinical trust by clarifying AI model predictions. Collaboration among researchers, clinicians, and patients ensures that research remains patient-centered and clinically relevant. Adapting advanced techniques for resource-limited settings through simplified workflows, open-source tools, and portable technologies democratizes access. Engaging local centers and training programs in underrepresented regions ensures diverse data and globally relevant findings.

Patient and public involvement (PPI) aligns research priorities with patient needs, enhancing relevance and impact [367]. For example, PPI in epilepsy trials highlighted overlooked mental health comorbidities [368]. Addressing scalability and inclusivity requires substantial investment and global collaboration. International consortiums like ENIGMA and the Human Brain Project exemplify the value of large-scale collaborations [369,370]. Enhancing reproducibility and clinical relevance necessitates strong validation structures and a better integration of diverse datasets. Establishing standardized pipelines for model validation can streamline the use of advanced tools like AI. Investments in low-cost iPSC platforms and AI-based computational models can democratize access to cutting-edge research tools. By synergizing computational and experimental approaches and cultivating strong collaborative frameworks, the field is poised to deliver more effective and personalized interventions, revolutionizing neuropsychiatric care.

## Figures and Tables

**Table 2:** Challenges and solutions in translating artificial intelligence (AI) models to clinics. AI, artificial intelligence; API, Application Programming Interface; EHR, electronic health record; XAI, explainable artificial intelligence; FDA, United States Food and Drug Administration; GPT, Generative Pre-trained Transformer; LIME, Local Interpretable Model Agnostic Explanation; ML, machine learning; NLP, natural language processing; SHAP, SHapley Additive exPlanations; XAI; explainable artificial intelligence.

Challenge	Description	Example/Context	Proposed Solution	Future Implications
Data Bias	Limited diversity in datasets leads to models that perform poorly across populations.	Neuroimaging datasets over-represent individuals from developed countries.	Collect data from underrepresented populations and build balanced datasets.	Improved model generalizability and equitable healthcare.
Lack of Interpretability	AI models, particularly deep learning, are often “black boxes,” making decisions hard to explain.	Clinical decisions influenced by opaque ML predictions.	Implement XAI techniques, such as SHAP or LIME frameworks.	Builds clinician trust and facilitates regulatory approval.
Scalability	High computational demands and infrastructure requirements restrict widespread adoption.	Training advanced models like GPT-based NLP systems.	Optimize algorithms and leverage cloud computing or edge AI technologies.	Reduces costs and enhances accessibility for smaller clinics.
Regulatory Barriers	Slow adaptation of regulatory frameworks for AI integration in clinical workflows.	FDA approval processes for AI tools in diagnostics.	Develop standardized guidelines and real-world evidence collection protocols.	Accelerates AI implementation in healthcare systems.
Data Privacy Concerns	Sensitive patient information is vulnerable to misuse or breaches during data collection and analysis.	Sharing genomic data for psychiatric biomarker research.	Use federated learning and encrypted data-sharing protocols.	Ensures secure collaboration without compromising privacy.
Validation and Reproducibility	AI models often lack external validation and reproducibility across clinical settings.	AI-based neuroimaging biomarkers not validated in multi-site trials.	Conduct multi-site, cross-population validation studies.	Increases confidence in clinical utility and robustness.
Integration with Existing Systems	AI tools often face challenges integrating with legacy EHR systems.	AI models for patient stratification requiring manual data input.	Develop interoperable APIs and adopt standardized data exchange formats.	Seamless AI adoption into routine clinical workflows.
Ethical Concerns	Potential for AI misuse, such as bias amplification or unfair treatment recommendations.	Disparities in AI-driven mental health treatment outcomes.	Implement ethical AI design principles and multidisciplinary oversight boards.	Ensures ethical and responsible deployment of AI.

**Table 3:** Key artificial intelligence (AI) applications in neuropsychiatric research. AI, artificial intelligence; AD, Alzheimer’s disease; BP, bipolar disorder; ML, machine language; NLP, natural language processing; SCZ, schizophrenia.

Application	Methodology	Outcome	Challenges	Future Directions	Reference
**Neuroimaging Biomarkers**	Deep Learning Models	Early detection and diagnosis of AD and SCZ	Data bias and limited generalizability	Use diverse training datasets; implement explainable AI	[276,277,278,279,280]
**Drug Discovery**	Predictive Modeling	Identification of novel compounds and drug repurposing	Lack of experimental validation pipelines	Develop AI-driven validation platforms using human-derived organoids	[281,282,283,284]
**Personalized Therapy**	Patient Stratification Models	Tailored treatment recommendations for depression and BP	Difficulty in accounting for multi-modal patient data	Integrate multi-omics and real-time patient monitoring data	[244,285,286,287,288]
**Disease Progression Prediction**	Temporal ML Models	Forecast disease stages and response to treatments	Overfitting due to limited long-term datasets	Establish longitudinal cohort studies with wearable sensors	[91,276,289,290,291]
**Mental Health Screening**	Natural Language Processing (NLP)	Automated analysis of patient speech and text for early mental illness detection	Privacy concerns and interpretability	Develop privacy-preserving algorithms and user-consent frameworks	[292,293,294,295,296]

## Data Availability

Data sharing is not applicable to this article.

## Abbreviations

The following abbreviations are used in this manuscript:

AD	Alzheimer’s disease
AI	artificial intelligence
BP	bipolar disorder
DSM-5	Diagnostic and Statistical Manual of Mental Disorders, Fifth Edition
GWAS	genome-wide association studies
HiTOP	Hierarchical Taxonomy of Psychopathology
iPSCs	Induced pluripotent stem cells
ML	machine learning
ncRNA	non-coding RNA
PPI	patient and public involvement
RDoC	Research Domain Criteria
SCZ	schizophrenia

## References

[1] Pepys M.B. (2007). Science and serendipity. Clin. Med..

[2] Li T., Vedula S.S., Hadar N., Parkin C., Lau J., Dickersin K. (2015). Innovations in data collection, management, and archiving for systematic reviews. Ann. Intern. Med..

[3] Liu Y., Qin C., Ma X., Liang H. (2022). Serendipity in human information behavior: A systematic review. J. Doc..

[4] Meyers M.A. (2011). Happy Accidents: Serendipity in Major Medical Breakthroughs in the Twentieth Century.

[5] Pievani T. (2024). Serendipity: The Unexpected in Science.

[6] Bauer M. (2020). Lithium: About discrepancies between efficacy and clinical use. Acta Psychiatr. Scand..

[7] Zarate C.A., Brutsche N.E., Ibrahim L., Franco-Chaves J., Diazgranados N., Cravchik A., Selter J., Marquardt C.A., Liberty V., Luckenbaugh D.A. (2012). Replication of ketamine’s antidepressant efficacy in bipolar depression: A randomized controlled add-on trial. Biol. Psychiatry.

[8] Smoller J.W. (2014). Psychiatric genetics and the future of personalized treatment. Depress. Anxiety.

[9] Rappa L.R., Larose-Pierre M., Branch E., Iglesias A.J., Norwood D.A., Simon W.A. (2001). Desperately seeking serendipity: The past, present, and future of antidepressant therapy. J. Pharm. Pract..

[10] Nutt D. (2014). Help luck along to find psychiatric medicines. Nature.

[11] Sharma A. (2016). Inflammatory and immune responses in depression. Curr. Neuropharmacol..

[12] McMahon F.J. (2014). Prediction of treatment outcomes in psychiatry—Where do we stand?. Dialogues Clin. Neurosci..

[13] Vaudano E. (2018). Public–private partnerships as enablers of progress in the fight against mental disorders: The example of the European Innovative Medicines Initiative. Eur. Psychiatry.

[14] Chekroud A.M., Bondar J., Delgadillo J., Doherty G., Wasil A., Fokkema M., Cohen Z., Belgrave D., DeRubeis R., Iniesta R. (2021). The promise of machine learning in predicting treatment outcomes in psychiatry. World Psychiatry.

[15] Kessler R.C., Luedtke A. (2021). Pragmatic precision psychiatry—A new direction for optimizing treatment selection. JAMA Psychiatry.

[16] Millan M.J., Goodwin G.M., Meyer-Lindenberg A., Ögren S.O. (2015). Learning from the past and looking to the future: Emerging perspectives for improving the treatment of psychiatric disorders. Eur. Neuropsychopharmacol..

[17] Tanaka M., Vécsei L. (2024). A Decade of Dedication: Pioneering Perspectives on Neurological Diseases and Mental Illnesses. Biomedicines.

[18] Leichsenring F., Steinert C., Rabung S., Ioannidis J.P. (2022). The efficacy of psychotherapies and pharmacotherapies for mental disorders in adults: An umbrella review and meta-analytic evaluation of recent meta-analyses. World Psychiatry.

[19] Marx W., Moseley G., Berk M., Jacka F. (2017). Nutritional psychiatry: The present state of the evidence. Proc. Nutr. Soc..

[20] Pesci N.R., Peracchia S., Teobaldi E., Maina G., Rosso G. (2024). Impact of mean monthly temperature on psychiatric admissions: Data from an acute inpatient unit. Eur. Psychiatry.

[21] Pieper A.A., Baraban J.M. (2017). Moving beyond serendipity to mechanism-driven psychiatric therapeutics. Neurotherapeutics.

[22] Ban T.A. (2006). The role of serendipity in drug discovery. Dialogues Clin. Neurosci..

[23] Punjabi P.P. (2018). Serendipity and margin of safety. Perfusion.

[24] Campbell W.C. (2005). Serendipity and new drugs for infectious disease. ILAR J..

[25] Đurić L., Milanović M., Milošević N., Milić N. (2020). New pharmaceuticals: The importance of serendipity. Med. Časopis.

[26] Jeste D.V., Gillin J.C., Wyatt R.J. (1979). Serendipity in biological psychiatry—A myth?. Arch. Gen. Psychiatry.

[27] Sverdlov O., Ryeznik Y., Wong W.K. (2021). Opportunity for efficiency in clinical development: An overview of adaptive clinical trial designs and innovative machine learning tools, with examples from the cardiovascular field. Contemp. Clin. Trials.

[28] Barkal J., Poi M., Dalton W. (2020). Abstract IA27: An innovative approach to improve clinical trials using adaptive in silico design. Cancer Epidemiol. Biomark. Prev..

[29] Wolkenhauer O., Auffray C., Jaster R., Steinhoff G., Dammann O. (2013). The road from systems biology to systems medicine. Pediatr. Res..

[30] Winter N.R., Blanke J., Leenings R., Ernsting J., Fisch L., Sarink K., Barkhau C., Emden D., Thiel K., Flinkenflügel K. (2024). A Systematic Evaluation of Machine Learning–Based Biomarkers for Major Depressive Disorder. JAMA Psychiatry.

[31] Di Camillo F., Grimaldi D.A., Cattarinussi G., Di Giorgio A., Locatelli C., Khuntia A., Enrico P., Brambilla P., Koutsouleris N., Sambataro F. (2024). Magnetic resonance imaging–based machine learning classification of schizophrenia spectrum disorders: A meta-analysis. Psychiatry Clin. Neurosci..

[32] Abi-Dargham A., Moeller S.J., Ali F., DeLorenzo C., Domschke K., Horga G., Jutla A., Kotov R., Paulus M.P., Rubio J.M. (2023). Candidate biomarkers in psychiatric disorders: State of the field. World Psychiatry.

[33] Cifci D., Foersch S., Kather J.N. (2022). Artificial intelligence to identify genetic alterations in conventional histopathology. J. Pathol..

[34] Ofek E., Haj R., Molchanov Y., Yacobi R., Mayer C., Barliya T., Gazy I., Dvir A., Hayun I., Zalach J. (2023). High-confidence AI-based biomarker profiling for H&E slides to optimize pathology workflow in lung cancer. J. Clin. Oncol..

[35] Bera K., Schalper K.A., Rimm D.L., Velcheti V., Madabhushi A. (2019). Artificial intelligence in digital pathology—New tools for diagnosis and precision oncology. Nat. Rev. Clin. Oncol..

[36] Salvi M., Acharya M.R., Seoni S., Faust O., Tan R.S., Barua P.D., García S., Molinari F., Acharya U.R. (2024). Artificial intelligence for atrial fibrillation detection, prediction, and treatment: A systematic review of the last decade (2013–2023). Wiley Interdiscip. Rev. Data Min. Knowl. Discov..

[37] Manetas-Stavrakakis N., Sotiropoulou I.M., Paraskevas T., Maneta Stavrakaki S., Bampatsias D., Xanthopoulos A., Papageorgiou N., Briasoulis A. (2023). Accuracy of artificial intelligence-based technologies for the diagnosis of atrial fibrillation: A systematic review and meta-analysis. J. Clin. Med..

[38] Calhoun V.D., Pearlson G.D., Sui J. (2021). Data-driven approaches to neuroimaging biomarkers for neurological and psychiatric disorders: Emerging approaches and examples. Curr. Opin. Neurol..

[39] Wolfers T., Buitelaar J.K., Beckmann C.F., Franke B., Marquand A.F. (2015). From estimating activation locality to predicting disorder: A review of pattern recognition for neuroimaging-based psychiatric diagnostics. Neurosci. Biobehav. Rev..

[40] Fonseka T.M., MacQueen G.M., Kennedy S.H. (2018). Neuroimaging biomarkers as predictors of treatment outcome in major depressive disorder. J. Affect. Disord..

[41] Papageorgiou I.E. (2023). Neuroscience Scaffolded by Informatics: A Raging Interdisciplinary Field. Symmetry.

[42] Mirmohammadi H., Fahmy M.D., Bidabadi F.S., Liang H. (2024). Editorial Letter: Breaking Down Boundaries: Unleashing the Power of Interdisciplinary Research. Sci. Hypotheses.

[43] Doom T., Raymer M., Krane D., Garcia O. (2003). Crossing the interdisciplinary barrier: A baccalaureate computer science option in bioinformatics. IEEE Trans. Educ..

[44] Logan S., Arzua T., Canfield S.G., Seminary E.R., Sison S.L., Ebert A.D., Bai X. (2019). Studying human neurological disorders using induced pluripotent stem cells: From 2D monolayer to 3D organoid and blood brain barrier models. Compr. Physiol..

[45] Aboul-Soud M.A., Alzahrani A.J., Mahmoud A. (2021). Induced pluripotent stem cells (iPSCs)—Roles in regenerative therapies, disease modelling and drug screening. Cells.

[46] Ho B.X., Pek N.M.Q., Soh B.-S. (2018). Disease modeling using 3D organoids derived from human induced pluripotent stem cells. Int. J. Mol. Sci..

[47] Karagiannis P., Takahashi K., Saito M., Yoshida Y., Okita K., Watanabe A., Inoue H., Yamashita J.K., Todani M., Nakagawa M. (2019). Induced pluripotent stem cells and their use in human models of disease and development. Physiol. Rev..

[48] Marchetto M.C., Brennand K.J., Boyer L.F., Gage F.H. (2011). Induced pluripotent stem cells (iPSCs) and neurological disease modeling: Progress and promises. Hum. Mol. Genet..

[49] Beevers J.E., Caffrey T.M., Wade-Martins R. (2013). Induced pluripotent stem cell (iPSC)-derived dopaminergic models of Parkinson’s disease. Biochem. Soc. Trans..

[50] Nguyen R., Bae S.D.W., Qiao L., George J. (2021). Developing liver organoids from induced pluripotent stem cells (iPSCs): An alternative source of organoid generation for liver cancer research. Cancer Lett..

[51] Trillhaase A., Maertens M., Aherrahrou Z., Erdmann J. (2021). Induced pluripotent stem cells (iPSCs) in vascular research: From two-to three-dimensional organoids. Stem Cell Rev. Rep..

[52] Wörheide M.A., Krumsiek J., Kastenmüller G., Arnold M. (2021). Multi-omics integration in biomedical research–A metabolomics-centric review. Anal. Chim. Acta.

[53] Sanches P.H.G., de Melo N.C., Porcari A.M., de Carvalho L.M. (2024). Integrating Molecular Perspectives: Strategies for Comprehensive Multi-Omics Integrative Data Analysis and Machine Learning Applications in Transcriptomics, Proteomics, and Metabolomics. Biology.

[54] Ge H., Walhout A.J., Vidal M. (2003). Integrating ‘omic’information: A bridge between genomics and systems biology. TRENDS Genet..

[55] Menyhárt O., Győrffy B. (2021). Multi-omics approaches in cancer research with applications in tumor subtyping, prognosis, and diagnosis. Comput. Struct. Biotechnol. J..

[56] Zhang B., Kuster B. (2019). Proteomics is not an island: Multi-omics integration is the key to understanding biological systems. Mol. Cell. Proteom..

[57] Song Y., Xu X., Wang W., Tian T., Zhu Z., Yang C. (2019). Single cell transcriptomics: Moving towards multi-omics. Analyst.

[58] Graw S., Chappell K., Washam C.L., Gies A., Bird J., Robeson M.S., Byrum S.D. (2021). Multi-omics data integration considerations and study design for biological systems and disease. Mol. Omics.

[59] Jendoubi T. (2021). Approaches to integrating metabolomics and multi-omics data: A primer. Metabolites.

[60] Pozhar K.V., Chuprakov D.A., Strukova E.I., Litinskaia E.L. Modeling of blood glucose dynamics to automate personalized insulin therapy for patients with type 1 diabetes mellitus. Proceedings of the 2023 IEEE Ural-Siberian Conference on Biomedical Engineering, Radioelectronics and Information Technology (USBEREIT).

[61] Wang Q., Molenaar P., Harsh S., Freeman K., Xie J., Gold C., Rovine M., Ulbrecht J. (2014). Personalized state-space modeling of glucose dynamics for type 1 diabetes using continuously monitored glucose, insulin dose, and meal intake: An extended Kalman filter approach. J. Diabetes Sci. Technol..

[62] Fan Y.N., Fan B., Lim C., Lau E.S., Tsoi S.T., Wan R., Lai W.Y., Poon E.W., Ho J., Ho C.C.Y. (2024). Precision Medicine to Redefine Insulin Secretion and Monogenic Diabetes-Randomized Controlled Trial (PRISM-RCT) in Chinese patients with young-onset diabetes: Design, methods and baseline characteristics. BMJ Open Diabetes Res. Care.

[63] Emery P., Dörner T. (2011). Optimising treatment in rheumatoid arthritis: A review of potential biological markers of response. Ann. Rheum. Dis..

[64] Takahashi S., Saegusa J., Onishi A., Morinobu A. (2019). Biomarkers identified by serum metabolomic analysis to predict biologic treatment response in rheumatoid arthritis patients. Rheumatology.

[65] Choi I.Y., Gerlag D.M., Herenius M.J., Thurlings R.M., Wijbrandts C.A., Foell D., Vogl T., Roth J., Tak P.P., Holzinger D. (2015). MRP8/14 serum levels as a strong predictor of response to biological treatments in patients with rheumatoid arthritis. Ann. Rheum. Dis..

[66] Saxe G.N., Statnikov A., Fenyo D., Ren J., Li Z., Prasad M., Wall D., Bergman N., Briggs E.C., Aliferis C. (2016). A complex systems approach to causal discovery in psychiatry. PLoS ONE.

[67] Nelson B., McGorry P.D., Wichers M., Wigman J.T., Hartmann J.A. (2017). Moving from static to dynamic models of the onset of mental disorder: A review. JAMA Psychiatry.

[68] Hernández R.M., Ponce-Meza J.C., Saavedra-López M.Á., Ugaz W.A.C., Chanduvi R.M., Monteza W.C. (2023). Brain Complexity and Psychiatric Disorders. Iran. J. Psychiatry.

[69] Zhao Y., Castellanos F.X. (2016). Annual research review: Discovery science strategies in studies of the pathophysiology of child and adolescent psychiatric disorders-promises and limitations. J. Child Psychol. Psychiatry.

[70] Totah N., Akil H., Huys Q.J., Krystal J.H., MacDonald A.W., Maia T.V., Malenka R.C., Pauli W.M. (2016). Complexity and Heterogeneity in Psychiatric Disorders: Opportunities for Computational Psychiatry. Computational Psychiatry: New Perspectives on Mental Illness.

[71] Gibbs R.M., Lipnick S., Bateman J.W., Chen L., Cousins H.C., Hubbard E.G., Jowett G., LaPointe D.S., McGredy M.J., Odonkor M.N. (2018). Toward precision medicine for neurological and neuropsychiatric disorders. Cell Stem Cell.

[72] Wen Z., Christian K.M., Song H., Ming G.-l. (2016). Modeling psychiatric disorders with patient-derived iPSCs. Curr. Opin. Neurobiol..

[73] Rashid B., Calhoun V. (2020). Towards a brain-based predictome of mental illness. Hum. Brain Mapp..

[74] Nicora G., Vitali F., Dagliati A., Geifman N., Bellazzi R. (2020). Integrated multi-omics analyses in oncology: A review of machine learning methods and tools. Front. Oncol..

[75] Demirel H.C., Arici M.K., Tuncbag N. (2022). Computational approaches leveraging integrated connections of multi-omic data toward clinical applications. Mol. Omics.

[76] Das S., Lecours Boucher X., Rogers C., Makowski C., Chouinard-Decorte F., Oros Klein K., Beck N., Rioux P., Brown S.T., Mohaddes Z. (2018). Integration of “omics” Data and phenotypic data within a unified extensible multimodal framework. Front. Neuroinformatics.

[77] Wu Y., Xie L. (2024). AI-driven multi-omics integration for multi-scale predictive modeling of causal genotype-environment-phenotype relationships. arXiv.

[78] Chan Y.H., Wang C., Soh W.K., Rajapakse J.C. (2022). Combining neuroimaging and omics datasets for disease classification using graph neural networks. Front. Neurosci..

[79] Wang H. (2024). The Application and Progress of Deep Learning in Bioinformatics. Comput. Mol. Biol..

[80] Vahabi N., Michailidis G. (2022). Unsupervised multi-omics data integration methods: A comprehensive review. Front. Genet..

[81] Chai H., Zhou X., Zhang Z., Rao J., Zhao H., Yang Y. (2021). Integrating multi-omics data through deep learning for accurate cancer prognosis prediction. Comput. Biol. Med..

[82] Gauld C., Depannemaecker D. (2023). Dynamical systems in computational psychiatry: A toy-model to apprehend the dynamics of psychiatric symptoms. Front. Psychol..

[83] Frank B., Jacobson N.C., Hurley L., McKay D. (2017). A theoretical and empirical modeling of anxiety integrated with RDoC and temporal dynamics. J. Anxiety Disord..

[84] Scheffer M., Bockting C.L., Borsboom D., Cools R., Delecroix C., Hartmann J.A., Kendler K.S., van de Leemput I., van der Maas H.L., van Nes E. (2024). A Dynamical Systems View of Psychiatric Disorders—Practical Implications: A Review. JAMA Psychiatry.

[85] Scheffer M., Bockting C.L., Borsboom D., Cools R., Delecroix C., Hartmann J.A., Kendler K.S., van de Leemput I., van der Maas H.L., van Nes E. (2024). A dynamical systems view of psychiatric disorders—Theory: A review. JAMA Psychiatry.

[86] Shin J.K., Malone D.T., Crosby I.T., Capuano B. (2011). Schizophrenia: A systematic review of the disease state, current therapeutics and their molecular mechanisms of action. Curr. Med. Chem..

[87] Meijboom F.L., Kostrzewa E., Leenaars C.H. (2020). Joining forces: The need to combine science and ethics to address problems of validity and translation in neuropsychiatry research using animal models. Philos. Ethics Humanit. Med..

[88] Thorp H.H. (2024). Bridging two views of autism. Science.

[89] Kozler P., Marešová D., Pokorný J. (2022). Determination of brain water content by dry/wet weight measurement for the detection of experimental brain edema. Physiol. Res..

[90] Benrimoh D.A., Friston K.J. (2020). All grown up: Computational theories of psychosis, complexity, and progress. J. Abnorm. Psychol..

[91] Ambrosen K.S., Skjerbæk M.W., Foldager J., Axelsen M.C., Bak N., Arvastson L., Christensen S.R., Johansen L.B., Raghava J.M., Oranje B. (2020). A machine-learning framework for robust and reliable prediction of short-and long-term treatment response in initially antipsychotic-naïve schizophrenia patients based on multimodal neuropsychiatric data. Transl. Psychiatry.

[92] Li L., Song C., Ma Y., Zou Y. (2023). “Half-wet-half-dry”: An innovation in undergraduate laboratory classes to generate transgenic mouse models using CRISPR/Cas9 and computer simulation. J. Biol. Educ..

[93] Nelson B., Lavoie S., Li E., Sass L., Koren D., McGorry P., Jack B., Parnas J., Polari A., Allott K. (2020). The neurophenomenology of early psychosis: An integrative empirical study. Conscious. Cogn..

[94] Yao S., Zhu J., Li S., Zhang R., Zhao J., Yang X., Wang Y. (2022). Bibliometric analysis of quantitative electroencephalogram research in neuropsychiatric disorders from 2000 to 2021. Front. Psychiatry.

[95] Huang H.-H., Li J., Cho W.C. (2023). Integrative analysis for complex disease biomarker discovery. Front. Bioeng. Biotechnol..

[96] Agarwal D., Marques G., de la Torre-Díez I., Franco Martin M.A., García Zapiraín B., Martín Rodríguez F. (2021). Transfer learning for Alzheimer’s disease through neuroimaging biomarkers: A systematic review. Sensors.

[97] Nyatega C.O., Qiang L., Adamu M.J., Kawuwa H.B. (2022). Gray matter, white matter and cerebrospinal fluid abnormalities in Parkinson’s disease: A voxel-based morphometry study. Front. Psychiatry.

[98] Younis A., Qiang L., Nyatega C.O., Adamu M.J., Kawuwa H.B. (2022). Brain tumor analysis using deep learning and VGG-16 ensembling learning approaches. Appl. Sci..

[99] de Lima E.P., Tanaka M., Lamas C.B., Quesada K., Detregiachi C.R.P., Araújo A.C., Guiguer E.L., Catharin V.M.C.S., de Castro M.V.M., Junior E.B. (2024). Vascular Impairment, Muscle Atrophy, and Cognitive Decline: Critical Age-Related Conditions. Biomedicines.

[100] Nunes Y.C., Mendes N.M., Pereira de Lima E., Chehadi A.C., Lamas C.B., Haber J.F., dos Santos Bueno M., Araújo A.C., Catharin V.C.S., Detregiachi C.R.P. (2024). Curcumin: A golden approach to healthy aging: A systematic review of the evidence. Nutrients.

[101] Tanaka M., Tuka B., Vécsei L. (2024). Navigating the Neurobiology of Migraine: From pathways to potential therapies. Cells.

[102] Mirkin S., Albensi B.C. (2023). Should artificial intelligence be used in conjunction with Neuroimaging in the diagnosis of Alzheimer’s disease?. Front. Aging Neurosci..

[103] Tilahun B.B.S., Thompson N.R., Sankary L.R., Laryea F., Trunick C.M., Jehi L.E. (2021). Outcomes in the treatment of psychogenic nonepileptic seizures (PNES) with CBTip: Response in seizure frequency, depression, anxiety, and quality of life. Epilepsy Behav..

[104] Velani H., Gledhill J. (2021). Psychological & Behavioural Treatments of Nonepileptic Seizures in Children and Adolescents. BJPsych Open.

[105] Aziz M.O., Mehrinejad S.A., Hashemian K., Paivastegar M. (2020). Integrative therapy (short-term psychodynamic psychotherapy & cognitive-behavioral therapy) and cognitive-behavioral therapy in the treatment of generalized anxiety disorder: A randomized controlled trial. Complement. Ther. Clin. Pract..

[106] Hall M.-F.E., Church F.C. (2020). Integrative medicine and health therapy for Parkinson disease. Top. Geriatr. Rehabil..

[107] Church F.C. (2021). Treatment options for motor and non-motor symptoms of Parkinson’s disease. Biomolecules.

[108] Nguyen S.A., Oughli H.A., Lavretsky H. (2024). Use of complementary and integrative medicine for Alzheimer’s disease and cognitive decline. J. Alzheimer’s Dis..

[109] Tanaka M., Vécsei L. (2024). Revolutionizing our understanding of Parkinson’s disease: Dr. Heinz Reichmann’s pioneering research and future research direction. J. Neural Transm..

[110] Pagotto G.L.d.O., Santos L.M.O.d., Osman N., Lamas C.B., Laurindo L.F., Pomini K.T., Guissoni L.M., Lima E.P.d., Goulart R.d.A., Catharin V.M.S. (2024). Ginkgo biloba: A Leaf of Hope in the Fight against Alzheimer’s Dementia: Clinical Trial Systematic Review. Antioxidants.

[111] Burnett S.D., Blanchette A.D., Chiu W.A., Rusyn I. (2021). Human induced pluripotent stem cell (iPSC)-derived cardiomyocytes as an in vitro model in toxicology: Strengths and weaknesses for hazard identification and risk characterization. Expert Opin. Drug Metab. Toxicol..

[112] Marcoux P., Hwang J.W., Desterke C., Imeri J., Bennaceur-Griscelli A., Turhan A.G. (2023). Modeling RET-Rearranged Non-Small Cell Lung Cancer (NSCLC): Generation of Lung Progenitor Cells (LPCs) from Patient-Derived Induced Pluripotent Stem Cells (iPSCs). Cells.

[113] Tanaka M., Vécsei L. (2024). From Lab to Life: Exploring Cutting-Edge Models for Neurological and Psychiatric Disorders. Biomedicines.

[114] Ho S.Y., Phua K., Wong L., Bin Goh W.W. (2020). Extensions of the External Validation for Checking Learned Model Interpretability and Generalizability. Patterns.

[115] Paolini Sguazzi G., Muto V., Tartaglia M., Bertini E., Compagnucci C. (2021). Induced Pluripotent Stem Cells (iPSCs) and Gene Therapy: A New Era for the Treatment of Neurological Diseases. Int. J. Mol. Sci..

[116] Yao X., Glessner J.T., Li J., Qi X., Hou X., Zhu C., Li X., March M.E., Yang L., Mentch F.D. (2021). Integrative analysis of genome-wide association studies identifies novel loci associated with neuropsychiatric disorders. Transl. Psychiatry.

[117] Mallard T.T., Grotzinger A.D., Smoller J.W. (2023). Examining the shared etiology of psychopathology with genome-wide association studies. Physiol. Rev..

[118] Eyring K.W., Geschwind D.H. (2021). Three decades of ASD genetics: Building a foundation for neurobiological understanding and treatment. Hum. Mol. Genet..

[119] Schwartzentruber J., Cooper S., Liu J.Z., Barrio-Hernandez I., Bello E., Kumasaka N., Young A.M.H., Franklin R.J.M., Johnson T., Estrada K. (2021). Genome-wide meta-analysis, fine-mapping and integrative prioritization implicate new Alzheimer’s disease risk genes. Nat. Genet..

[120] Dalmasso M.C., de Rojas I., Olivar N., Muchnik C., Angel B., Gloger S., Sanchez Abalos M.S., Chacón M.V., Aránguiz R., Orellana P. (2024). The first genome-wide association study in the Argentinian and Chilean populations identifies shared genetics with Europeans in Alzheimer’s disease. Alzheimers Dement..

[121] Andrews S.J., Fulton-Howard B., Goate A. (2020). Interpretation of risk loci from genome-wide association studies of Alzheimer’s disease. Lancet Neurol..

[122] Uffelmann E., Posthuma D. (2021). Emerging Methods and Resources for Biological Interrogation of Neuropsychiatric Polygenic Signal. Biol. Psychiatry.

[123] Hernandez L.M., Kim M., Hoftman G.D., Haney J.R., de la Torre-Ubieta L., Pasaniuc B., Gandal M.J. (2021). Transcriptomic Insight Into the Polygenic Mechanisms Underlying Psychiatric Disorders. Biol. Psychiatry.

[124] Gedik H., Nguyen T.H., Peterson R.E., Chatzinakos C., Vladimirov V.I., Riley B.P., Bacanu S.A. (2023). Identifying potential risk genes and pathways for neuropsychiatric and substance use disorders using intermediate molecular mediator information. Front. Genet..

[125] Yao Y., Guo W., Zhang S., Yu H., Yan H., Zhang H., Sanders A.R., Yue W., Duan J. (2021). Cell type-specific and cross-population polygenic risk score analyses of MIR137 gene pathway in schizophrenia. iScience.

[126] Kibinge N.K., Relton C.L., Gaunt T.R., Richardson T.G. (2020). Characterizing the Causal Pathway for Genetic Variants Associated with Neurological Phenotypes Using Human Brain-Derived Proteome Data. Am. J. Hum. Genet..

[127] Schwarz E.L., Pegolotti L., Pfaller M.R., Marsden A.L. (2023). Beyond CFD: Emerging methodologies for predictive simulation in cardiovascular health and disease. Biophys. Rev..

[128] Hirschhorn M., Tchantchaleishvili V., Stevens R., Rossano J., Throckmorton A. (2020). Fluid-structure interaction modeling in cardiovascular medicine—A systematic review 2017-2019. Med. Eng. Phys..

[129] Cluitmans M., Walton R., Plank G. (2023). Editorial: Computational methods in cardiac electrophysiology. Front. Physiol..

[130] Ramachandran R.P., Akbarzadeh M., Paliwal J., Cenkowski S. (2018). Computational fluid dynamics in drying process modelling—A technical review. Food Bioprocess Technol..

[131] Defraeye T. (2014). Advanced computational modelling for drying processes–A review. Appl. Energy.

[132] Duruflé H., Selmani M., Ranocha P., Jamet E., Dunand C., Déjean S. (2021). A powerful framework for an integrative study with heterogeneous omics data: From univariate statistics to multi-block analysis. Brief. Bioinform..

[133] Reel P.S., Reel S., Pearson E., Trucco E., Jefferson E. (2021). Using machine learning approaches for multi-omics data analysis: A review. Biotechnol. Adv..

[134] Bodein A., Scott-Boyer M.P., Perin O., Lê Cao K.A., Droit A. (2022). Interpretation of network-based integration from multi-omics longitudinal data. Nucleic Acids Res..

[135] Bhattacharya A., Li Y., Love M.I. (2021). MOSTWAS: Multi-Omic Strategies for Transcriptome-Wide Association Studies. PLoS Genet..

[136] Akiyama M. (2021). Multi-omics study for interpretation of genome-wide association study. J. Hum. Genet..

[137] Paczkowska M., Barenboim J., Sintupisut N., Fox N.S., Zhu H., Abd-Rabbo D., Mee M.W., Boutros P.C., Reimand J. (2020). Integrative pathway enrichment analysis of multivariate omics data. Nat. Commun..

[138] Kawuwa H.B., Nyatega C.O., Younis A., Adamu M.J. (2024). Neuroanatomical alterations in brain disorder: A magnetic resonance imaging analysis. Int. J. Sci. Res. Arch..

[139] Adamu M.J., Qiang L., Nyatega C.O., Younis A., Kawuwa H.B., Jabire A.H., Saminu S. (2023). Unraveling the pathophysiology of schizophrenia: Insights from structural magnetic resonance imaging studies. Front. Psychiatry.

[140] Kourou K., Exarchos T.P., Exarchos K.P., Karamouzis M.V., Fotiadis D.I. (2015). Machine learning applications in cancer prognosis and prediction. Comput. Struct. Biotechnol. J..

[141] Battaglia S., Nazzi C., Fullana M.A., di Pellegrino G., Borgomaneri S. (2024). ‘Nip it in the bud’: Low-frequency rTMS of the prefrontal cortex disrupts threat memory consolidation in humans. Behav. Res. Ther..

[142] Battineni G., Sagaro G.G., Chinatalapudi N., Amenta F. (2020). Applications of Machine Learning Predictive Models in the Chronic Disease Diagnosis. J. Pers. Med..

[143] El Naqa I., Bradley J.D., Lindsay P.E., Hope A.J., Deasy J.O. (2009). Predicting radiotherapy outcomes using statistical learning techniques. Phys. Med. Biol..

[144] Liang Z., Verkhivker G.M., Hu G. (2020). Integration of network models and evolutionary analysis into high-throughput modeling of protein dynamics and allosteric regulation: Theory, tools and applications. Brief. Bioinform..

[145] Huang N.F., Chaudhuri O., Cahan P., Wang A., Engler A.J., Wang Y., Kumar S., Khademhosseini A., Li S. (2020). Multi-scale cellular engineering: From molecules to organ-on-a-chip. APL Bioeng..

[146] John-Herpin A., Kavungal D., von Mücke L., Altug H. (2021). Infrared Metasurface Augmented by Deep Learning for Monitoring Dynamics between All Major Classes of Biomolecules. Adv. Mater..

[147] Battaglia S., Avenanti A., Vécsei L., Tanaka M. (2024). Neural correlates and molecular mechanisms of memory and learning. Int. J. Mol. Sci..

[148] Quettier T., Ippolito G., Però L., Cardellicchio P., Battaglia S., Borgomaneri S. (2024). Individual differences in intracortical inhibition predict action control when facing emotional stimuli. Front. Psychol..

[149] Fakhri S., Darvish E., Narimani F., Moradi S.Z., Abbaszadeh F., Khan H. (2023). The regulatory role of non-coding RNAs and their interactions with phytochemicals in neurodegenerative diseases: A systematic review. Brief. Funct. Genom..

[150] Brennan G.P., Henshall D.C. (2020). MicroRNAs as regulators of brain function and targets for treatment of epilepsy. Nat. Rev. Neurol..

[151] Rajendra P., Brahmajirao V. (2020). Modeling of dynamical systems through deep learning. Biophys. Rev..

[152] Terranova N., Venkatakrishnan K. (2024). Machine Learning in Modeling Disease Trajectory and Treatment Outcomes: An Emerging Enabler for Model-Informed Precision Medicine. Clin. Pharmacol. Ther..

[153] Koumakis L. (2020). Deep learning models in genomics; are we there yet?. Comput. Struct. Biotechnol. J..

[154] Watson D.S. (2022). Interpretable machine learning for genomics. Hum. Genet..

[155] Martínez-García M., Hernández-Lemus E. (2021). Data Integration Challenges for Machine Learning in Precision Medicine. Front. Med..

[156] Wright J.T., Herzberg M.C. (2021). Science for the Next Century: Deep Phenotyping. J. Dent. Res..

[157] Schalkamp A.K., Rahman N., Monzón-Sandoval J., Sandor C. (2022). Deep phenotyping for precision medicine in Parkinson’s disease. Dis. Model. Mech..

[158] Bourgeais V., Zehraoui F., Ben Hamdoune M., Hanczar B. (2021). Deep GONet: Self-explainable deep neural network based on Gene Ontology for phenotype prediction from gene expression data. BMC Bioinform..

[159] Liu M., Shen X., Pan W. (2022). Deep reinforcement learning for personalized treatment recommendation. Stat. Med..

[160] Chang C.Y., Ting H.C., Liu C.A., Su H.L., Chiou T.W., Lin S.Z., Harn H.J., Ho T.J. (2020). Induced Pluripotent Stem Cell (iPSC)-Based Neurodegenerative Disease Models for Phenotype Recapitulation and Drug Screening. Molecules.

[161] Jusop A.S., Thanaskody K., Tye G.J., Dass S.A., Wan Kamarul Zaman W.S., Nordin F. (2023). Development of brain organoid technology derived from iPSC for the neurodegenerative disease modelling: A glance through. Front. Mol. Neurosci..

[162] Valadez-Barba V., Cota-Coronado A., Hernández-Pérez O.R., Lugo-Fabres P.H., Padilla-Camberos E., Díaz N.F., Díaz-Martínez N.E. (2020). iPSC for modeling neurodegenerative disorders. Regen. Ther..

[163] Qian L., Tcw J. (2021). Human iPSC-Based Modeling of Central Nerve System Disorders for Drug Discovery. Int. J. Mol. Sci..

[164] Pomeshchik Y., Klementieva O., Gil J., Martinsson I., Hansen M.G., de Vries T., Sancho-Balsells A., Russ K., Savchenko E., Collin A. (2020). Human iPSC-Derived Hippocampal Spheroids: An Innovative Tool for Stratifying Alzheimer Disease Patient-Specific Cellular Phenotypes and Developing Therapies. Stem Cell Rep..

[165] Trombetta-Lima M., Sabogal-Guáqueta A.M., Dolga A.M. (2021). Mitochondrial dysfunction in neurodegenerative diseases: A focus on iPSC-derived neuronal models. Cell Calcium.

[166] Amponsah A.E., Guo R., Kong D., Feng B., He J., Zhang W., Liu X., Du X., Ma Z., Liu B. (2021). Patient-derived iPSCs, a reliable in vitro model for the investigation of Alzheimer’s disease. Rev. Neurosci..

[167] Li J., Fraenkel E. (2021). Phenotyping Neurodegeneration in Human iPSCs. Annu. Rev. Biomed. Data Sci..

[168] Hyman S.E. (2021). Use of mouse models to investigate the contributions of CNVs associated with schizophrenia and autism to disease mechanisms. Curr. Opin. Genet. Dev..

[169] Neuhaus C.P. (2022). Threats to Benefits: Assessing Knowledge Production in Nonhuman Models of Human Neuropsychiatric Disorders. Hastings Cent. Rep..

[170] Voikar V., Gaburro S. (2020). Three Pillars of Automated Home-Cage Phenotyping of Mice: Novel Findings, Refinement, and Reproducibility Based on Literature and Experience. Front. Behav. Neurosci..

[171] Palmer D., Dumont J.R., Dexter T.D., Prado M.A.M., Finger E., Bussey T.J., Saksida L.M. (2021). Touchscreen cognitive testing: Cross-species translation and co-clinical trials in neurodegenerative and neuropsychiatric disease. Neurobiol. Learn. Mem..

[172] Winiarski M., Kondrakiewicz L., Kondrakiewicz K., Jędrzejewska-Szmek J., Turzyński K., Knapska E., Meyza K. (2022). Social deficits in BTBR T+ Itpr3tf/J mice vary with ecological validity of the test. Genes. Brain Behav..

[173] Cwiek A., Rajtmajer S.M., Wyble B., Honavar V., Grossner E., Hillary F.G. (2022). Feeding the machine: Challenges to reproducible predictive modeling in resting-state connectomics. Netw. Neurosci..

[174] Naaktgeboren C.A., Ochodo E.A., Van Enst W.A., de Groot J.A.H., Hooft L., Leeflang M.M.G., Bossuyt P.M., Moons K.G.M., Reitsma J.B. (2016). Assessing variability in results in systematic reviews of diagnostic studies. BMC Med. Res. Methodol..

[175] Rasero J., Sentis A.I., Yeh F.C., Verstynen T. (2021). Integrating across neuroimaging modalities boosts prediction accuracy of cognitive ability. PLoS Comput. Biol..

[176] Jiang R., Woo C.W., Qi S., Wu J., Sui J. (2022). Interpreting Brain Biomarkers: Challenges and solutions in interpreting machine learning-based predictive neuroimaging. IEEE Signal Process Mag..

[177] Kohoutová L., Heo J., Cha S., Lee S., Moon T., Wager T.D., Woo C.W. (2020). Toward a unified framework for interpreting machine-learning models in neuroimaging. Nat. Protoc..

[178] Eitel F., Schulz M.A., Seiler M., Walter H., Ritter K. (2021). Promises and pitfalls of deep neural networks in neuroimaging-based psychiatric research. Exp. Neurol..

[179] Wachinger C., Rieckmann A., Pölsterl S. (2021). Detect and correct bias in multi-site neuroimaging datasets. Med. Image Anal..

[180] Sui J., Jiang R., Bustillo J., Calhoun V. (2020). Neuroimaging-based Individualized Prediction of Cognition and Behavior for Mental Disorders and Health: Methods and Promises. Biol. Psychiatry.

[181] Vaden K.I., Gebregziabher M., Dyslexia Data C., Eckert M.A. (2020). Fully synthetic neuroimaging data for replication and exploration. Neuroimage.

[182] Saha D.K., Calhoun V.D., Du Y., Fu Z., Kwon S.M., Sarwate A.D., Panta S.R., Plis S.M. (2022). Privacy-preserving quality control of neuroimaging datasets in federated environments. Hum. Brain Mapp..

[183] Mohammed Yakubu A., Chen Y.P. (2020). Ensuring privacy and security of genomic data and functionalities. Brief Bioinform..

[184] Tanaka M., Battaglia S., Giménez-Llort L., Chen C., Hepsomali P., Avenanti A., Vécsei L. (2024). Innovation at the intersection: Emerging translational research in neurology and psychiatry. Cells.

[185] Panov G., Panova P. (2024). Neurobiochemical disturbances in psychosis and their implications for therapeutic intervention. Curr. Top. Med. Chem..

[186] Bonanno M., Calabrò R.S. (2023). Bridging the Gap between Basic Research and Clinical Practice: The Growing Role of Translational Neurorehabilitation. Medicines.

[187] Heider J., Vogel S., Volkmer H., Breitmeyer R. (2021). Human iPSC-Derived Glia as a Tool for Neuropsychiatric Research and Drug Development. Int. J. Mol. Sci..

[188] Lakiotaki K., Papadovasilakis Z., Lagani V., Fafalios S., Charonyktakis P., Tsagris M., Tsamardinos I. (2023). Automated machine learning for genome wide association studies. Bioinformatics.

[189] Xiao Q., Bai X., Zhang C., He Y. (2022). Advanced high-throughput plant phenotyping techniques for genome-wide association studies: A review. J. Adv. Res..

[190] Reynolds T., Johnson E.C., Huggett S.B., Bubier J.A., Palmer R.H.C., Agrawal A., Baker E.J., Chesler E.J. (2021). Interpretation of psychiatric genome-wide association studies with multispecies heterogeneous functional genomic data integration. Neuropsychopharmacology.

[191] McGill M.P., Threadgill D.W. (2023). Adding robustness to rigor and reproducibility for the three Rs of improving translational medical research. J. Clin. Investig..

[192] Schubert R., Geoffroy E., Gregga I., Mulford A.J., Aguet F., Ardlie K., Gerszten R., Clish C., Van Den Berg D., Taylor K.D. (2022). Protein prediction for trait mapping in diverse populations. PLoS ONE.

[193] Sesia M., Bates S., Candès E., Marchini J., Sabatti C. (2021). False discovery rate control in genome-wide association studies with population structure. Proc. Natl. Acad. Sci. USA.

[194] Wegner C.D., Mount B.A., Colvis C.M. (2022). A public-private collaboration model for clinical innovation. Clin. Transl. Sci..

[195] Vogel A.L., Knebel A.R., Faupel-Badger J.M., Portilla L.M., Simeonov A. (2021). A systems approach to enable effective team science from the internal research program of the National Center for Advancing Translational Sciences. J. Clin. Transl. Sci..

[196] Becich M.J. (2023). Clinical Trial Strategies Fueled by Informatics Innovation Catalyze Translational Research. JAMA Netw. Open.

[197] Munro C.L., Savel R.H. (2017). Rigor and Reproducibility in Critical Care Research. Am. J. Crit. Care.

[198] Díaz-Faes A.A., Llopis O., D’Este P., Molas-Gallart J. (2023). Assessing the variety of collaborative practices in translational research: An analysis of scientists’ ego-networks. Res. Eval..

[199] Lenze E.J., Nicol G.E., Barbour D.L., Kannampallil T., Wong A.W.K., Piccirillo J., Drysdale A.T., Sylvester C.M., Haddad R., Miller J.P. (2021). Precision clinical trials: A framework for getting to precision medicine for neurobehavioural disorders. J. Psychiatry Neurosci..

[200] Nabbout R., Kuchenbuch M. (2020). Impact of predictive, preventive and precision medicine strategies in epilepsy. Nat. Rev. Neurol..

[201] Rees E., Owen M.J. (2020). Translating insights from neuropsychiatric genetics and genomics for precision psychiatry. Genome Med..

[202] Salazar de Pablo G., Studerus E., Vaquerizo-Serrano J., Irving J., Catalan A., Oliver D., Baldwin H., Danese A., Fazel S., Steyerberg E.W. (2021). Implementing Precision Psychiatry: A Systematic Review of Individualized Prediction Models for Clinical Practice. Schizophr. Bull..

[203] Alciati A., Reggiani A., Caldirola D., Perna G. (2022). Human-Induced Pluripotent Stem Cell Technology: Toward the Future of Personalized Psychiatry. J. Pers. Med..

[204] Battaglia S., Avenanti A., Vécsei L., Tanaka M. (2024). Neurodegeneration in cognitive impairment and mood disorders for experimental, clinical and translational neuropsychiatry. Biomedicines.

[205] Jester D.J., Thomas M.L., Sturm E.T., Harvey P.D., Keshavan M., Davis B.J., Saxena S., Tampi R., Leutwyler H., Compton M.T. (2023). Review of Major Social Determinants of Health in Schizophrenia-Spectrum Psychotic Disorders: I. Clinical Outcomes. Schizophr. Bull..

[206] Panov G. (2022). Gender-associated role in patients with schizophrenia. Is there a connection with the resistance?. Front. Psychiatry.

[207] Panov G., Dyulgerova S., Panova P., Stefanova S. (2024). Untangling Depression in Schizophrenia: The Role of Disorganized and Obsessive-Compulsive Symptoms and the Duration of Untreated Psychosis. Biomedicines.

[208] Panov G., Dyulgerova S., Panova P. (2023). Cognition in Patients with Schizophrenia: Interplay between Working Memory, Disorganized Symptoms, Dissociation, and the Onset and Duration of Psychosis, as Well as Resistance to Treatment. Biomedicines.

[209] Panov G., Panova P. (2023). Obsessive-compulsive symptoms in patient with schizophrenia: The influence of disorganized symptoms, duration of schizophrenia, and drug resistance. Front. Psychiatry.

[210] Blackwell M.A., Goodkind J.R., Yeater E.A., Van Horn M.L. (2024). Predictors of mental health outcomes of three refugee groups in an advocacy-based intervention: A precision medicine perspective. J. Consult. Clin. Psychol..

[211] Beaudoin M., Hudon A., Giguère C.E., Potvin S., Dumais A. (2022). Prediction of quality of life in schizophrenia using machine learning models on data from Clinical Antipsychotic Trials of Intervention Effectiveness (CATIE) schizophrenia trial. Schizophrenia.

[212] Gaeta A.M., Quijada-López M., Barbé F., Vaca R., Pujol M., Minguez O., Sánchez-de-la-Torre M., Muñoz-Barrutia A., Piñol-Ripoll G. (2024). Predicting Alzheimer’s disease CSF core biomarkers: A multimodal Machine Learning approach. Front. Aging Neurosci..

[213] Shim M., Lee S.H., Hwang H.J. (2021). Inflated prediction accuracy of neuropsychiatric biomarkers caused by data leakage in feature selection. Sci. Rep..

[214] Davatzikos C., Barnholtz-Sloan J.S., Bakas S., Colen R., Mahajan A., Quintero C.B., Capellades Font J., Puig J., Jain R., Sloan A.E. (2020). AI-based prognostic imaging biomarkers for precision neuro-oncology: The ReSPOND consortium. Neuro Oncol..

[215] Khanna N.N., Maindarkar M.A., Viswanathan V., Puvvula A., Paul S., Bhagawati M., Ahluwalia P., Ruzsa Z., Sharma A., Kolluri R. (2022). Cardiovascular/Stroke Risk Stratification in Diabetic Foot Infection Patients Using Deep Learning-Based Artificial Intelligence: An Investigative Study. J. Clin. Med..

[216] Ben-Naim S., Dienstag A., Freedman S.A., Ekstein D., Foul Y.A., Gilad M., Peled O., Waldman A., Oster S., Azoulay M. (2020). A Novel Integrative Psychotherapy for Psychogenic Nonepileptic Seizures Based on the Biopsychosocial Model: A Retrospective Pilot Outcome Study. Psychosomatics.

[217] Cobb S.J., Vaughn B.V., Sagherian K. (2023). Nonpharmacologic Interventions and Seizure Frequency in Patients With Psychogenic Nonepileptic Seizures: An Integrative Review. J. Am. Psychiatr. Nurses Assoc..

[218] Jeyaraman M., Balaji S., Jeyaraman N., Yadav S. (2023). Unraveling the Ethical Enigma: Artificial Intelligence in Healthcare. Cureus.

[219] Angehrn Z., Sostar J., Nordon C., Turner A., Gove D., Karcher H., Keenan A., Mittelstadt B., de Reydet-de Vulpillieres F. (2020). Ethical and Social Implications of Using Predictive Modeling for Alzheimer’s Disease Prevention: A Systematic Literature Review. J. Alzheimers Dis..

[220] Larson D.B., Magnus D.C., Lungren M.P., Shah N.H., Langlotz C.P. (2020). Ethics of Using and Sharing Clinical Imaging Data for Artificial Intelligence: A Proposed Framework. Radiology.

[221] Kassam I., Ilkina D., Kemp J., Roble H., Carter-Langford A., Shen N. (2023). Patient Perspectives and Preferences for Consent in the Digital Health Context: State-of-the-art Literature Review. J. Med. Internet Res..

[222] Yarborough B.J.H., Stumbo S.P. (2023). A Stakeholder-Informed Ethical Framework to Guide Implementation of Suicide Risk Prediction Models Derived from Electronic Health Records. Arch. Suicide Res..

[223] Liang X., Zhao J., Chen Y., Bandara E., Shetty S. (2023). Architectural Design of a Blockchain-Enabled, Federated Learning Platform for Algorithmic Fairness in Predictive Health Care: Design Science Study. J. Med. Internet Res..

[224] Bear Don’t Walk O.J.t., Reyes Nieva H., Lee S.S., Elhadad N. (2022). A scoping review of ethics considerations in clinical natural language processing. JAMIA Open.

[225] Haggarty S.J., Karmacharya R., Perlis R.H. (2021). Advances toward precision medicine for bipolar disorder: Mechanisms & molecules. Mol. Psychiatry.

[226] Malhi G.S., Outhred T. (2016). Therapeutic mechanisms of lithium in bipolar disorder: Recent advances and current understanding. CNS Drugs.

[227] Kavalali E.T., Monteggia L.M. (2020). Targeting homeostatic synaptic plasticity for treatment of mood disorders. Neuron.

[228] Gao T.-H., Ni R.-J., Liu S., Tian Y., Wei J., Zhao L., Wang Q., Ni P., Ma X., Li T. (2021). Chronic lithium exposure attenuates ketamine-induced mania-like behavior and c-Fos expression in the forebrain of mice. Pharmacol. Biochem. Behav..

[229] Scott J., Etain B., Bellivier F. (2018). Can an integrated science approach to precision medicine research improve lithium treatment in bipolar disorders?. Front. Psychiatry.

[230] Nasrallah H.A. (2012). The hazards of serendipity. Curr. Psychiatry.

[231] Nestler E.J., Gould E., Manji H. (2002). Preclinical models: Status of basic research in depression. Biol. Psychiatry.

[232] Hayashi-Takagi A., Araki Y., Nakamura M., Vollrath B., Duron S.G., Yan Z., Kasai H., Huganir R.L., Campbell D.A., Sawa A. (2014). PAKs inhibitors ameliorate schizophrenia-associated dendritic spine deterioration in vitro and in vivo during late adolescence. Proc. Natl. Acad. Sci. USA.

[233] Papakostas G., Ionescu D. (2015). Towards new mechanisms: An update on therapeutics for treatment-resistant major depressive disorder. Mol. Psychiatry.

[234] Hartl D., de Luca V., Kostikova A., Laramie J., Kennedy S., Ferrero E., Siegel R., Fink M., Ahmed S., Millholland J. (2021). Translational precision medicine: An industry perspective. J. Transl. Med..

[235] Gandal M.J., Leppa V., Won H., Parikshak N.N., Geschwind D.H. (2016). The road to precision psychiatry: Translating genetics into disease mechanisms. Nat. Neurosci..

[236] Alzoubi L., Aljabali A.A.A., Tambuwala M.M. (2023). Empowering Precision Medicine: The Impact of 3D Printing on Personalized Therapeutic. AAPS PharmSciTech..

[237] Srinivasan N., Mehra E., Dommaraju S., Kakavetsis E. (2024). Neurogenetics: Precision Medicine-Based Approaches to Neurological Disorders with an Emphasis on Addressing Alzheimer’s Disease and Schizophrenia. Berkeley Pharma Tech. J. Med..

[238] Mumtaz H., Saqib M., Jabeen S., Muneeb M., Mughal W., Sohail H., Safdar M., Mehmood Q., Khan M.A., Ismail S.M. (2023). Exploring alternative approaches to precision medicine through genomics and artificial intelligence–a systematic review. Front. Med..

[239] Chang E.H., Zabner J. (2015). Precision genomic medicine in cystic fibrosis. Clin. Transl. Sci..

[240] Fanen P., Wohlhuter-Haddad A., Hinzpeter A. (2014). Genetics of cystic fibrosis: CFTR mutation classifications toward genotype-based CF therapies. Int. J. Biochem. Cell Biol..

[241] Southern K.W., Murphy J., Sinha I.P., Nevitt S.J. (2020). Corrector therapies (with or without potentiators) for people with cystic fibrosis with class II CFTR gene variants (most commonly F508del). Cochrane Database Syst. Rev..

[242] Rosenquist R., Bernard E., Erkers T., Scott D.W., Itzykson R., Rousselot P., Soulier J., Hutchings M., Östling P., Cavelier L. (2023). Novel precision medicine approaches and treatment strategies in hematological malignancies. J. Intern. Med..

[243] Takei T., Yokoyama K., Yusa N., Nakamura S., Ogawa M., Kondoh K., Kobayashi M., Kobayashi A., Ito M., Shimizu E. (2018). Artificial Intelligence Guided Precision Medicine Approach to Hematological Disease. Blood.

[244] Uddin M., Wang Y., Woodbury-Smith M. (2019). Artificial intelligence for precision medicine in neurodevelopmental disorders. NPJ Digit. Med..

[245] Marques L., Costa B., Pereira M., Silva A., Santos J., Saldanha L., Silva I., Magalhães P., Schmidt S., Vale N. (2024). Advancing precision medicine: A review of innovative In Silico approaches for drug development, clinical pharmacology and personalized healthcare. Pharmaceutics.

[246] Kuch D., Kearnes M., Gulson K. (2020). The promise of precision: Datafication in medicine, agriculture and education. Policy Stud..

[247] Cirillo D., Valencia A. (2019). Big data analytics for personalized medicine. Curr. Opin. Biotechnol..

[248] Nedungadi P., Iyer A., Gutjahr G., Bhaskar J., Pillai A.B. (2018). Data-driven methods for advancing precision oncology. Curr. Pharmacol. Rep..

[249] Kosorok M.R., Laber E.B. (2019). Precision medicine. Annu. Rev. Stat. Its Appl..

[250] Ahmed Z. (2022). Multi-omics strategies for personalized and predictive medicine: Past, current, and future translational opportunities. Emerg. Top. Life Sci..

[251] Agur Z., Elishmereni M., Foryś U., Kogan Y. (2020). Accelerating the development of personalized cancer immunotherapy by integrating molecular patients’ profiles with dynamic mathematical models. Clin. Pharmacol. Ther..

[252] Prosperi M., Min J.S., Bian J., Modave F. (2018). Big data hurdles in precision medicine and precision public health. BMC Med. Inform. Decis. Mak..

[253] Cuthbert B.N. (2014). The RDoC framework: Facilitating transition from ICD/DSM to dimensional approaches that integrate neuroscience and psychopathology. World Psychiatry.

[254] Cuthbert B.N., Insel T.R. (2013). Toward the future of psychiatric diagnosis: The seven pillars of RDoC. BMC Med..

[255] Tanaka M. (2024). Beyond the boundaries: Transitioning from categorical to dimensional paradigms in mental health diagnostics. Adv. Clin. Exp. Med..

[256] Clark L.A., Cuthbert B., Lewis-Fernández R., Narrow W.E., Reed G.M. (2017). Three approaches to understanding and classifying mental disorder: ICD-11, DSM-5, and the National Institute of Mental Health’s Research Domain Criteria (RDoC). Psychol. Sci. Public. Interest..

[257] Möller H.J., Bandelow B., Bauer M., Hampel H., Herpertz S.C., Soyka M., Barnikol U.B., Lista S., Severus E., Maier W. (2015). DSM-5 reviewed from different angles: Goal attainment, rationality, use of evidence, consequences—Part 2: Bipolar disorders, schizophrenia spectrum disorders, anxiety disorders, obsessive-compulsive disorders, trauma- and stressor-related disorders, personality disorders, substance-related and addictive disorders, neurocognitive disorders. Eur. Arch. Psychiatry Clin. Neurosci..

[258] Lilienfeld S.O., Treadway M.T. (2016). Clashing Diagnostic Approaches: DSM-ICD Versus RDoC. Annu. Rev. Clin. Psychol..

[259] Fusar-Poli P., Solmi M., Brondino N., Davies C., Chae C., Politi P., Borgwardt S., Lawrie S.M., Parnas J., McGuire P. (2019). Transdiagnostic psychiatry: A systematic review. World Psychiatry.

[260] Carcone D., Ruocco A.C. (2017). Six years of research on the national institute of mental health’s research domain criteria (RDoC) initiative: A systematic review. Front. Cell. Neurosci..

[261] Kelly J., Clarke G., Cryan J., Dinan T. (2018). Dimensional thinking in psychiatry in the era of the Research Domain Criteria (RDoC). Ir. J. Psychol. Med..

[262] Knudsen E.I. (2007). Fundamental components of attention. Annu. Rev. Neurosci..

[263] Michelini G., Palumbo I.M., DeYoung C.G., Latzman R.D., Kotov R. (2021). Linking RDoC and HiTOP: A new interface for advancing psychiatric nosology and neuroscience. Clin. Psychol. Rev..

[264] Pacheco J., Garvey M.A., Sarampote C.S., Cohen E.D., Murphy E.R., Friedman-Hill S.R. (2022). Annual Research Review: The contributions of the RDoC research framework on understanding the neurodevelopmental origins, progression and treatment of mental illnesses. J. Child Psychol. Psychiatry.

[265] Rezapour T., Rafei P., Baldacchino A., Conrod P.J., Dom G., Fishbein D.H., Kazemi A., Hendriks V., Newton N., Riggs N.R. (2024). Neuroscience-informed classification of prevention interventions in substance use disorders: An RDoC-based approach. Neurosci. Biobehav. Rev..

[266] Manchia M., Pisanu C., Squassina A., Carpiniello B. (2020). Challenges and future prospects of precision medicine in psychiatry. Pharmacogenomics Pers. Med..

[267] DeLisi L.E., Fleischhacker W.W. (2016). How precise is precision medicine for schizophrenia?. Curr. Opin. Psychiatry.

[268] Wamsley B., Geschwind D.H. (2020). Functional genomics links genetic origins to pathophysiology in neurodegenerative and neuropsychiatric disease. Curr. Opin. Genet. Dev..

[269] Lago S.G., Tomasik J., van Rees G.F., Ramsey J.M., Haenisch F., Cooper J.D., Broek J.A., Suarez-Pinilla P., Ruland T., Auyeug B. (2020). Exploring the neuropsychiatric spectrum using high-content functional analysis of single-cell signaling networks. Mol. Psychiatry.

[270] Goud Alladi C., Etain B., Bellivier F., Marie-Claire C. (2018). DNA methylation as a biomarker of treatment response variability in serious mental illnesses: A systematic review focused on bipolar disorder, schizophrenia, and major depressive disorder. Int. J. Mol. Sci..

[271] Hollander J.A., Cory-Slechta D.A., Jacka F.N., Szabo S.T., Guilarte T.R., Bilbo S.D., Mattingly C.J., Moy S.S., Haroon E., Hornig M. (2020). Beyond the looking glass: Recent advances in understanding the impact of environmental exposures on neuropsychiatric disease. Neuropsychopharmacology.

[272] Fries G.R. (2019). Genetics and epigenetics as tools to inform the pathophysiology of neuropsychiatric disorders. Braz. J. Psychiatry.

[273] van de Leemput J., Glatt S.J., Tsuang M.T. (2016). The potential of genetic and gene expression analysis in the diagnosis of neuropsychiatric disorders. Expert Rev. Mol. Diagn..

[274] Soliman M., Aboharb F., Zeltner N., Studer L. (2017). Pluripotent stem cells in neuropsychiatric disorders. Mol. Psychiatry.

[275] Magwai T., Oginga F.O., Chiliza B., Mpofana T., Xulu K.R. (2022). Genome-wide DNA methylation in an animal model and human studies of schizophrenia: A protocol for a meta-analysis. BMJ Open Sci..

[276] Shah J., Rahman Siddiquee M.M., Krell-Roesch J., Syrjanen J.A., Kremers W.K., Vassilaki M., Forzani E., Wu T., Geda Y.E. (2023). Neuropsychiatric Symptoms and Commonly Used Biomarkers of Alzheimer’s Disease: A Literature Review from a Machine Learning Perspective. J. Alzheimer’s Dis..

[277] Jo T., Nho K., Saykin A.J. (2019). Deep learning in Alzheimer’s disease: Diagnostic classification and prognostic prediction using neuroimaging data. Front. Aging Neurosci..

[278] Etekochay M.O., Amaravadhi A.R., González G.V., Atanasov A.G., Matin M., Mofatteh M., Steinbusch H.W., Tesfaye T., Praticò D. (2024). Unveiling new strategies facilitating the implementation of artificial intelligence in neuroimaging for the early detection of Alzheimer’s disease. J. Alzheimer’s Dis..

[279] Du Y., Niu J., Xing Y., Li B., Calhoun V.D. (2024). Neuroimage Analysis Methods and Artificial Intelligence Techniques for Reliable Biomarkers and Accurate Diagnosis of Schizophrenia: Achievements Made by Chinese Scholars Around the Past Decade. Schizophr. Bull..

[280] Dong C., Hayashi S. (2024). Deep learning applications in vascular dementia using neuroimaging. Curr. Opin. Psychiatry.

[281] Costamagna G., Comi G.P., Corti S. (2021). Advancing drug discovery for neurological disorders using iPSC-derived neural organoids. Int. J. Mol. Sci..

[282] Vatansever S., Schlessinger A., Wacker D., Kaniskan H.Ü., Jin J., Zhou M.M., Zhang B. (2021). Artificial intelligence and machine learning-aided drug discovery in central nervous system diseases: State-of-the-arts and future directions. Med. Res. Rev..

[283] Bhattamisra S.K., Banerjee P., Gupta P., Mayuren J., Patra S., Candasamy M. (2023). Artificial intelligence in pharmaceutical and healthcare research. Big Data Cogn. Comput..

[284] Schöning V., Khurana A., Karolak A. (2023). Spotlight on artificial intelligence in experimental pharmacology and drug discovery. Front. Pharmacol..

[285] Tran B.X., McIntyre R.S., Latkin C.A., Phan H.T., Vu G.T., Nguyen H.L.T., Gwee K.K., Ho C.S., Ho R.C. (2019). The current research landscape on the artificial intelligence application in the management of depressive disorders: A bibliometric analysis. Int. J. Environ. Res. Public Health.

[286] Squarcina L., Villa F.M., Nobile M., Grisan E., Brambilla P. (2021). Deep learning for the prediction of treatment response in depression. J. Affect. Disord..

[287] Tornero-Costa R., Martinez-Millana A., Azzopardi-Muscat N., Lazeri L., Traver V., Novillo-Ortiz D. (2023). Methodological and quality flaws in the use of artificial intelligence in mental health research: Systematic review. JMIR Ment. Health.

[288] Yuan Y., Wu Y., Sui H., Deng R. Research on the application of Artificial Intelligence in Bipolar Disorder. Proceedings of the 2023 4th International Symposium on Artificial Intelligence for Medicine Science.

[289] Wang Y., Tang S., Ma R., Zamit I., Wei Y., Pan Y. (2022). Multi-modal intermediate integrative methods in neuropsychiatric disorders: A review. Comput. Struct. Biotechnol. J..

[290] Myszczynska M.A., Ojamies P.N., Lacoste A.M., Neil D., Saffari A., Mead R., Hautbergue G.M., Holbrook J.D., Ferraiuolo L. (2020). Applications of machine learning to diagnosis and treatment of neurodegenerative diseases. Nat. Rev. Neurol..

[291] Fisher C.K., Smith A.M., Walsh J.R. (2018). Deep learning for comprehensive forecasting of Alzheimer’s Disease progression. arXiv.

[292] Gerantia M. (2024). Artificial Intelligence in Psychiatry: A Comprehensive Literature Review. Eur. Psychiatry.

[293] Kerz E., Zanwar S., Qiao Y., Wiechmann D. (2023). Toward explainable AI (XAI) for mental health detection based on language behavior. Front. Psychiatry.

[294] Graham S., Depp C., Lee E.E., Nebeker C., Tu X., Kim H.-C., Jeste D.V. (2019). Artificial intelligence for mental health and mental illnesses: An overview. Curr. Psychiatry Rep..

[295] Biró A., Cuesta-Vargas A.I., Szilágyi L. (2023). Precognition of mental health and neurogenerative disorders using AI-parsed text and sentiment analysis. Acta Univ. Sapientiae Inform..

[296] Lovejoy C.A. (2019). Technology and mental health: The role of artificial intelligence. Eur. Psychiatry.

[297] Fernandes B.S., Karmakar C., Tamouza R., Tran T., Yearwood J., Hamdani N., Laouamri H., Richard J.-R., Yolken R., Berk M. (2020). Precision psychiatry with immunological and cognitive biomarkers: A multi-domain prediction for the diagnosis of bipolar disorder or schizophrenia using machine learning. Transl. Psychiatry.

[298] Sethi S., Brietzke E. (2016). Omics-based biomarkers: Application of metabolomics in neuropsychiatric disorders. Int. J. Neuropsychopharmacol..

[299] Hoehe M.R., Morris-Rosendahl D.J. (2018). The role of genetics and genomics in clinical psychiatry. Dialogues Clin. Neurosci..

[300] Abi-Dargham A., Horga G. (2016). The search for imaging biomarkers in psychiatric disorders. Nat. Med..

[301] Fu C.H., Costafreda S.G. (2013). Neuroimaging-based biomarkers in psychiatry: Clinical opportunities of a paradigm shift. Can. J. Psychiatry.

[302] Parnas J. (1999). From predisposition to psychosis: Progression of symptoms in schizophrenia. Acta Psychiatr. Scand..

[303] Cannon T.D., Van Erp T.G., Bearden C.E., Loewy R., Thompson P., Toga A.W., Huttunnen M.O., Keshavan M.S., Seidman L.J., Tsuang M.T. (2003). Early and late neurodevelopmental influences in the prodrome to schizophrenia: Contributions of genes, environment, and their interactions. Schizophr. Bull..

[304] Salokangas R.K., McGlashan T.H. (2008). Early detection and intervention of psychosis. A review. Nord. J. Psychiatry.

[305] Fakra E., Kaladjian A., Da Fonseca D., Maurel M., Adida M., Besnier N., Pringuey D., Azorin J.M. (2010). [Prodromal phase in bipolar disorder]. Encephale.

[306] Poletti M., Raballo A. (2020). Developmental Psychotic Risk: Toward a Neurodevelopmentally Informed Staging of Vulnerability to Psychosis. Harv. Rev. Psychiatry.

[307] Chen Z.S., Kulkarni P.P., Galatzer-Levy I.R., Bigio B., Nasca C., Zhang Y. (2022). Modern views of machine learning for precision psychiatry. Patterns.

[308] Tai A.M.Y., Albuquerque A., Carmona N.E., Subramanieapillai M., Cha D.S., Sheko M., Lee Y., Mansur R., McIntyre R.S. (2019). Machine learning and big data: Implications for disease modeling and therapeutic discovery in psychiatry. Artif. Intell. Med..

[309] Gifford G., Crossley N., Fusar-Poli P., Schnack H.G., Kahn R.S., Koutsouleris N., Cannon T.D., McGuire P. (2017). Using neuroimaging to help predict the onset of psychosis. Neuroimage.

[310] Bedi G., Carrillo F., Cecchi G.A., Slezak D.F., Sigman M., Mota N.B., Ribeiro S., Javitt D.C., Copelli M., Corcoran C.M. (2015). Automated analysis of free speech predicts psychosis onset in high-risk youths. NPJ Schizophr..

[311] Corcoran C.M., Carrillo F., Fernández-Slezak D., Bedi G., Klim C., Javitt D.C., Bearden C.E., Cecchi G.A. (2018). Prediction of psychosis across protocols and risk cohorts using automated language analysis. World Psychiatry.

[312] Del Casale A., Sarli G., Bargagna P., Polidori L., Alcibiade A., Zoppi T., Borro M., Gentile G., Zocchi C., Ferracuti S. (2023). Machine Learning and Pharmacogenomics at the Time of Precision Psychiatry. Curr. Neuropharmacol..

[313] Bracher-Smith M., Crawford K., Escott-Price V. (2021). Machine learning for genetic prediction of psychiatric disorders: A systematic review. Mol. Psychiatry.

[314] Alhuwaydi A.M. (2024). Exploring the Role of Artificial Intelligence in Mental Healthcare: Current Trends and Future Directions—A Narrative Review for a Comprehensive Insight. Risk Manag. Healthc. Policy.

[315] Ayhan Y. (2023). The Impact of Artificial Intelligence on Psychiatry: Benefits and Concerns-An essay from a disputed ‘author’. Turk. Psikiyatr. Derg..

[316] Cuthbert B.N. (2020). The role of RDoC in future classification of mental disorders. Dialogues Clin. Neurosci..

[317] Yager J., Feinstein R.E. (2017). Potential Applications of the National Institute of Mental Health’s Research Domain Criteria (RDoC) to Clinical Psychiatric Practice: How RDoC Might Be Used in Assessment, Diagnostic Processes, Case Formulation, Treatment Planning, and Clinical Notes. J. Clin. Psychiatry.

[318] Beauchaine T.P., Thayer J.F. (2015). Heart rate variability as a transdiagnostic biomarker of psychopathology. Int. J. Psychophysiol..

[319] Young J.J., Silber T., Bruno D., Galatzer-Levy I.R., Pomara N., Marmar C.R. (2016). Is there Progress? An Overview of Selecting Biomarker Candidates for Major Depressive Disorder. Front. Psychiatry.

[320] Hsin H., Fromer M., Peterson B., Walter C., Fleck M., Campbell A., Varghese P., Califf R. (2018). Transforming Psychiatry into Data-Driven Medicine with Digital Measurement Tools. NPJ Digit. Med..

[321] Scala J.J., Ganz A.B., Snyder M.P. (2023). Precision Medicine Approaches to Mental Health Care. Physiology.

[322] Roche D., Russell V. (2021). Can precision medicine advance psychiatry?. Ir. J. Psychol. Med..

[323] Parimbelli E., Marini S., Sacchi L., Bellazzi R. (2018). Patient similarity for precision medicine: A systematic review. J. Biomed. Inform..

[324] Lin E., Lin C.H., Lane H.Y. (2020). Precision Psychiatry Applications with Pharmacogenomics: Artificial Intelligence and Machine Learning Approaches. Int. J. Mol. Sci..

[325] Schwitzer T., Leboyer M., Laprévote V., Louis Dorr V., Schwan R. (2022). Using retinal electrophysiology toward precision psychiatry. Eur. Psychiatry.

[326] Schmaal L. (2023). The curse and opportunity of heterogeneity in the pursuit of psychiatric biomarkers. World Psychiatry..

[327] O’Halloran R., Kopell B.H., Sprooten E., Goodman W.K., Frangou S. (2016). Multimodal neuroimaging-informed clinical applications in neuropsychiatric disorders. Front. Psychiatry.

[328] Bansal R., Staib L.H., Laine A.F., Hao X., Xu D., Liu J., Weissman M., Peterson B.S. (2012). Anatomical brain images alone can accurately diagnose chronic neuropsychiatric illnesses. PLoS ONE.

[329] Striano P., Minassian B.A. (2020). From genetic testing to precision medicine in epilepsy. Neurotherapeutics.

[330] Lin M., Lachman H.M., Zheng D. (2016). Transcriptomics analysis of iPSC-derived neurons and modeling of neuropsychiatric disorders. Mol. Cell. Neurosci..

[331] Wen J., Skampardoni I., Tian Y.E., Yang Z., Cui Y., Erus G., Hwang G., Varol E., Boquet-Pujadas A., Chand G.B. (2024). Nine Neuroimaging-AI Endophenotypes Unravel Disease Heterogeneity and Partial Overlap across Four Brain Disorders: A Dimensional Neuroanatomical Representation. medRxiv.

[332] Whitfield-Gabrieli S., Ghosh S., Nieto-Castanon A., Saygin Z., Doehrmann O., Chai X., Reynolds G., Hofmann S., Pollack M., Gabrieli J. (2016). Brain connectomics predict response to treatment in social anxiety disorder. Mol. Psychiatry.

[333] Martin R.F., Leppink-Shands P., Tlachac M., DuBois M., Conelea C., Jacob S., Morellas V., Morris T., Papanikolopoulos N. (2021). The use of immersive environments for the early detection and treatment of neuropsychiatric disorders. Front. Digit. Health.

[334] Grezenko H., Rodoshi Z.N., Mimms C.S., Ahmed M., Sabani A., Hlaing M.S., Batu B.J., Hundesa M.I., Ayalew B.D., Shehryar A. (2024). From Alzheimer’s Disease to Anxiety, Epilepsy to Schizophrenia: A Comprehensive Dive Into Neuro-Psychiatric Disorders. Cureus.

[335] Kas M.J., Penninx B., Sommer B., Serretti A., Arango C., Marston H. (2019). A quantitative approach to neuropsychiatry: The why and the how. Neurosci. Biobehav. Rev..

[336] Malhi G.S., Sachdev P. (2002). Novel physical treatments for the management of neuropsychiatric disorders. J. Psychosom. Res..

[337] Berk M. (2012). Pathways to new drug discovery in neuropsychiatry. BMC Med..

[338] Gandal M.J., Geschwind D.H. (2016). The genetics-driven revival in neuropsychiatric drug development. Biol. Psychiatry.

[339] Spicer T.P., Hubbs C., Vaissiere T., Collia D., Rojas C., Kilinc M., Vick K., Madoux F., Baillargeon P., Shumate J. (2018). Improved scalability of neuron-based phenotypic screening assays for therapeutic discovery in neuropsychiatric disorders. Mol. Neuropsychiatry.

[340] Asgharian P., Quispe C., Herrera-Bravo J., Sabernavaei M., Hosseini K., Forouhandeh H., Ebrahimi T., Sharafi-Badr P., Tarhriz V., Soofiyani S.R. (2022). Pharmacological effects and therapeutic potential of natural compounds in neuropsychiatric disorders: An update. Front. Pharmacol..

[341] O’Donnell P., Rosen L., Alexander R., Murthy V., Davies C.H., Ratti E. (2019). Strategies to address challenges in neuroscience drug discovery and development. Int. J. Neuropsychopharmacol..

[342] Bearden C.E., Winkler A., Karlsgodt K.H., Bilder R. (2016). Cognitive phenotypes and endophenotypes: Concepts and criteria. Psychiatry and Neuropsychology in the “OMICS” Era.

[343] Hannan A.J. (2013). Nature, Nurture and neurobiology: Gene-environment interactions in neuropsychiatric disorders. Neurobiol. Dis..

[344] Willsey A.J., Morris M.T., Wang S., Willsey H.R., Sun N., Teerikorpi N., Baum T.B., Cagney G., Bender K.J., Desai T.A. (2018). The psychiatric cell map initiative: A convergent systems biological approach to illuminating key molecular pathways in neuropsychiatric disorders. Cell.

[345] Tropea D. (2012). New challenges and frontiers in the research for neuropsychiatric disorders. Front. Psychiatry.

[346] Sanders S.J., Sahin M., Hostyk J., Thurm A., Jacquemont S., Avillach P., Douard E., Martin C.L., Modi M.E., Moreno-De-Luca A. (2019). A framework for the investigation of rare genetic disorders in neuropsychiatry. Nat. Med..

[347] Dauncey M.J. (2013). Genomic and epigenomic insights into nutrition and brain disorders. Nutrients.

[348] Afridi R., Seol S., Kang H.J., Suk K. (2021). Brain-immune interactions in neuropsychiatric disorders: Lessons from transcriptome studies for molecular targeting. Biochem. Pharmacol..

[349] Alter O., Newman E., Ponnapalli S.P., Tsai J.W. (2024). AI/ML-derived mechanistically interpretable whole-genome biomarkers of patient survival in pre-treatment primary neuroblastoma tumors and whole blood. J. Clin. Oncol..

[350] Vadapalli S., Abdelhalim H., Zeeshan S., Ahmed Z. (2022). Artificial intelligence and machine learning approaches using gene expression and variant data for personalized medicine. Brief. Bioinform..

[351] Bello B., Bundey Y.N., Bhave R., Khotimchenko M., Baran S.W., Chakravarty K., Varshney J. (2023). Integrating AI/ML models for patient stratification leveraging omics dataset and clinical biomarkers from COVID-19 patients: A promising approach to personalized medicine. Int. J. Mol. Sci..

[352] Kobeissy F., Goli M., Yadikar H., Shakkour Z., Kurup M., Haidar M.A., Alroumi S., Mondello S., Wang K.K., Mechref Y. (2023). Advances in neuroproteomics for neurotrauma: Unraveling insights for personalized medicine and future prospects. Front. Neurol..

[353] Graham S.A., Lee E.E., Jeste D.V., Van Patten R., Twamley E.W., Nebeker C., Yamada Y., Kim H.-C., Depp C.A. (2020). Artificial intelligence approaches to predicting and detecting cognitive decline in older adults: A conceptual review. Psychiatry Res..

[354] Hsiao Y.-C., Dutta A. (2024). Network Modeling and Control of Dynamic Disease Pathways, Review and Perspectives. IEEE/ACM Trans. Comput. Biol. Bioinform..

[355] Fan X., Zhu P., Tang X.-Q. (2020). VD-analysis: A dynamic network framework for analyzing disease progressions. IEEE Access.

[356] Shmulevich I., Dougherty E.R., Zhang W. (2002). Gene perturbation and intervention in probabilistic Boolean networks. Bioinformatics.

[357] Perrone M.C., Lerner M.G., Dunworth M., Ewald A.J., Bader J.S. (2024). Prioritizing drug targets by perturbing biological network response functions. PLoS Comput. Biol..

[358] McGarry K., McDonald S. (2018). Complex network theory for the identification and assessment of candidate protein targets. Comput. Biol. Med..

[359] Cadeddu C., Ianuale C., Lindert J. (2015). Public mental health. A Systematic Review of Key Issues in Public Health.

[360] Singla D.R., Kohrt B.A., Murray L.K., Anand A., Chorpita B.F., Patel V. (2017). Psychological treatments for the world: Lessons from low-and middle-income countries. Annu. Rev. Clin. Psychol..

[361] Maresova P., Javanmardi E., Barakovic S., Barakovic Husic J., Tomsone S., Krejcar O., Kuca K. (2019). Consequences of chronic diseases and other limitations associated with old age - a scoping review. BMC Public Health.

[362] Agid Y., Buzsáki G., Diamond D.M., Frackowiak R., Giedd J., Girault J.-A., Grace A., Lambert J.J., Manji H., Mayberg H. (2007). How can drug discovery for psychiatric disorders be improved?. Nat. Rev. Drug Discov..

[363] Squassina A. (2021). Personalized treatments in neuropsychiatric disorders. Drug Dev. Res..

[364] Ilomäki J., Bell J.S., Chan A.Y., Tolppanen A.-M., Luo H., Wei L., Lai E.C.-C., Shin J.-Y., De Paoli G., Pajouheshnia R. (2020). Application of healthcare ‘Big Data’in CNS drug research: The example of the neurological and mental health Global Epidemiology Network (NeuroGEN). CNS Drugs.

[365] Vervoort I., Delger C., Soubry A. (2022). A multifactorial model for the etiology of neuropsychiatric disorders: The role of advanced paternal age. Pediatr. Res..

[366] Liloia D., Zamfira D.A., Tanaka M., Manuello J., Crocetta A., Keller R., Cozzolino M., Duca S., Cauda F., Costa T. (2024). Disentangling the role of gray matter volume and concentration in autism spectrum disorder: A meta-analytic investigation of 25 years of voxel-based morphometry research. Neurosci. Biobehav. Rev..

[367] Goulao B., Bruhn H., Campbell M., Ramsay C., Gillies K. (2021). Patient and public involvement in numerical aspects of trials (PoINT): Exploring patient and public partners experiences and identifying stakeholder priorities. Trials.

[368] Michaelis R., Tang V., Nevitt S.J., Wagner J.L., Modi A.C., LaFrance W.C., Goldstein L.H., Gandy M., Bresnahan R., Valente K. (2020). Psychological treatments for people with epilepsy. Cochrane Database Syst. Rev..

[369] Thompson P.M., Jahanshad N., Ching C.R.K., Salminen L.E., Thomopoulos S.I., Bright J., Baune B.T., Bertolín S., Bralten J., Bruin W.B. (2020). ENIGMA and global neuroscience: A decade of large-scale studies of the brain in health and disease across more than 40 countries. Transl. Psychiatry.

[370] Lorents A., Colin M.E., Bjerke I.E., Nougaret S., Montelisciani L., Diaz M., Verschure P., Vezoli J. (2023). Human Brain Project Partnering Projects Meeting: Status Quo and Outlook. eNeuro.

